# *Clostridium perfringens* chitinases, key enzymes during early stages of necrotic enteritis in broiler chickens

**DOI:** 10.1371/journal.ppat.1012560

**Published:** 2024-09-16

**Authors:** Evelien Dierick, Chana Callens, Yehudi Bloch, Savvas N. Savvides, Sarah Hark, Stefan Pelzer, Richard Ducatelle, Filip Van Immerseel, Evy Goossens

**Affiliations:** 1 Livestock Gut Health Team (LiGHT) Ghent, Department of Pathobiology, Pharmacology and Zoological Medicine, Faculty of Veterinary Medicine, Ghent University, Merelbeke, Belgium; 2 Unit for Structural Biology, Department of Biochemistry and Microbiology, Ghent University, Ghent, Belgium; 3 Unit for Structural Biology, VIB-UGent Center for Inflammation Research, Ghent, Belgium; 4 Current address: European Molecular Biology Laboratory, EMBL Hamburg, c/o DESY, Hamburg, Germany; 5 Evonik Operations GmbH, Nutrition & Care, Halle, Westfalen, Germany; National Institutes of Health, UNITED STATES OF AMERICA

## Abstract

The interaction between bacteria and the intestinal mucus is crucial during the early pathogenesis of many enteric diseases in mammals. A critical step in this process employed by both commensal and pathogenic bacteria focuses on the breakdown of the protective layer presented by the intestinal mucus by mucolytic enzymes. *C*. *perfringens* type G, the causative agent of necrotic enteritis in broilers, produces two glycosyl hydrolase family 18 chitinases, ChiA and ChiB, which display distinct substrate preferences. Whereas ChiB preferentially processes linear substrates such as chitin, ChiA prefers larger and more branched substrates, such as carbohydrates presented by the chicken intestinal mucus. Here, we show via crystal structures of ChiA and ChiB in the apo and ligand-bound forms that the two enzymes display structural features that explain their substrate preferences providing a structural blueprint for further interrogation of their function and inhibition. This research focusses on the roles of ChiA and ChiB in bacterial proliferation and mucosal attachment, two processes leading to colonization and invasion of the gut. ChiA and ChiB, either supplemented or produced by the bacteria, led to a significant increase in *C*. *perfringens* growth. In addition to nutrient acquisition, the importance of chitinases in bacterial attachment to the mucus layer was shown using an *in vitro* binding assay of *C*. *perfringens* to chicken intestinal mucus. Both an *in vivo* colonization trial and a necrotic enteritis trial were conducted, demonstrating that a ChiA chitinase mutant strain was less capable to colonize the intestine and was hampered in its disease-causing ability as compared to the wild-type strain. Our findings reveal that the pathogen-specific chitinases produced by *C*. *perfringens* type G strains play a fundamental role during colonization, suggesting their potential as vaccine targets.

## Author summary

The intestinal mucus layer protects the intestinal mucosa from invasion by pathogenic bacteria. However, it is precisely this first line of protection that invading bacteria aim to break down *en route* to colonizing the gut. *Clostridium perfringens* type G, the causative agent of necrotic enteritis in broiler chickens, an enteric disease characterised by ulcers in the small intestine, employs such a pathogenic strategy. In addition to the well-studied NetB toxin, this bacterium can produce other pathogen-specific enzymes, some of which could play a central role during disease development. Two of these enzymes were identified as putative chitinases, ChiA and ChiB. We have demonstrated that ChiA and ChiB are indeed functional enzymes that can hydrolyze a wide variety of substrates, with ChiA preferring mucus as a substrate. Mucus breakdown by the chitinases induces both bacterial proliferation and attachment to mucus, two processes that are crucial in the first steps of the pathogenesis. In addition, we have shown that a ChiA mutant strain does not efficiently colonize the intestine and causes less severe lesions as compared to the wild-type strain. Thus, we conclude that chitinases provide an advantage to the pathogen to facilitate colonization of the intestine of broiler chickens.

## Introduction

Glycans are ubiquitous throughout nature and play a fundamental role in many biological processes. These carbohydrate residues are diverse in structure and composition and can be part of glycoproteins, glycolipids or other glycoconjugates [1]. Throughout the gastrointestinal tract of vertebrates, glycans are omnipresent. First, the gut epithelium is covered with a mucus layer composed of highly glycosylated proteins, called mucins. This mucus layer acts as a first line of defence against food particles, gut microbiota, chemicals, enzymes and host or microbial secreted products, and contributes to the symbiosis between the host and the microbiota [2]. Furthermore, the host produces glycoproteins and glycolipids that are attached to the apical side of epithelial cells, called the glycocalyx, inhibiting bacterial adherence through steric hinderance [3]. Lastly, almost all crucial molecules involved in the innate and adaptive immune system are highly glycosylated [4]. Both direct and indirect functions of glycans in the immune system have been described, ranging from their action as ligands, immunogens, and antigens to other more complex processes like cell-cell recognition and antibody glycosylation [5].

The intestinal microbiota can utilize these glycans in different ways. Mucin glycans can be broken down to metabolizable oligomers that can act as carbon or energy sources [6]. Also, these carbohydrate structures can provide initial attachment sites for bacteria [7]. Pathogens have evolved to exploit this niche using the breakdown of or attachment to glycans to their advantage, aiding the initial steps of colonization and proliferation [8,9]. Furthermore, detection of glycan residues or monosaccharides in the environment can act as a chemical cue to help the pathogens sense their surroundings. As a response, pathogens can initiate the expression of virulence factors which may further impair the protective mucus layer, leading to colonization and eventually infection [6,9].

Both commensal and pathogenic bacteria are able to produce a large array of glycosyl hydrolases, enzymes that are able to cleave glycosidic bonds of glycans. Chitinases are glycosyl hydrolases that hydrolyze the β-(1–4)-linkage of N-acetyl-D-glucosamine (GlcNAc) units which are present amongst others in chitin, a linear polymer of GlcNAc. Chitinases are produced by many different bacteria and other life forms like plants, mammals, insects and fungi [10]. Bacterial chitinases are predominantly members of the glycosyl hydrolase families 18 or 19 [11]. Chitin is the second most abundant biopolymer in nature and forms the main component of fungal cell walls, crustacean shells and arthropod exoskeletons [12]. Vertebrates, with the exception of certain fish and amphibian species, do not have the ability to synthesize chitin [13,14]. Despite the lack of chitin in the intestinal tract, various enzymes and proteins which are annotated as chitinases and chitin-binding proteins have been linked to pathogenesis of enteric bacterial diseases [11]. Potential targets, other than chitin, are the β-1,4-linkage in GlcNAc-containing glycolipids and glycoproteins, which are omnipresent in the gastrointestinal tract [11,15]. Involvement of bacterial chitinases and chitin-binding proteins has been shown in mucus breakdown, bacterial translocation, suppression of host innate immune system and bacterial colonization through attachment to the intestinal epithelium, highlighting the crucial role of these enzymes as key virulence factors in a range of bacterial intestinal diseases [11,16–21].

*Clostridium perfringens* type G is the causative agent of necrotic enteritis (NE) in broilers, an enteric disease with great economic consequences [22]. *C*. *perfringens* is part of the normal gut microbiota of vertebrates, reaching numbers up to 10^5^ cfu/g in small intestinal content of healthy chickens [23]. Dysregulation of the intestinal microbiota can trigger proliferation of a virulent *C*. *perfringens* strain, eventually followed by massive toxin production. In contrast to non-pathogenic *C*. *perfringens* strains, the type G strains harbour three specific pathogenicity loci associated to their virulence potential: NELoc-1 (42kb; plasmid encoded), NELoc-2 (11.2kb; chromosomal) and NELoc-3 (5.6kb; plasmid- encoded) [24]. Using a *netB-*knockout mutant, the NetB toxin, located on NELoc-1, has been identified as a critical virulence factor in NE pathogenesis [25]. Deletion of the plasmid harbouring the entire NELoc-1 locus including the *netB* gene, results in complete loss of virulence, which is, however, only partially restored by complementation of this mutant with the *netB* gene, suggesting the importance of other potential virulence genes located on the plasmid [26]. In addition to the *netB* gene, NELoc-1 harbours an additional 36 genes from which two have been identified as putative chitinases, ChiA and ChiB. Interestingly, a study focussing on the *C*. *perfringens* gene expression during initial colonization of the intestinal tract, showed that both chitinase genes were upregulated during the early stages of pathogenesis, while expression of none of the 34 other genes located on the plasmid (incl. NetB) was increased, suggesting a possible involvement of these putative chitinases during colonization [27]. The aim of this research was to elucidate whether these chitinase genes encode functionally active proteins and to study their potential involvement during the early stages of NE pathogenesis. Structural studies were performed using X-ray crystallography, resulting in the description of the binding pockets and potential carbohydrate ligand interactions. *In vitro* binding studies as well as enzymatic activity studies were performed towards multiple substrates (pseudo-chitin substrates, chitin, mucus), thereby questioning the allocated nomenclature as true chitinases. To determine the biological relevance of chitinases, a combination of *in vitro* assays, either using *C*. *perfringens* mutant strains or recombinantly produced enzymes, were conducted studying the role of chitinases during either nutrient acquisition or bacterial binding to intestinal mucus. Finally, the reduced ability of a ChiA mutant strain to colonize the small intestine and induce NE was assessed using an *in vivo* colonization assay and NE challenge trial, respectively.

## Results

### Subcellular localization of *C*. *perfringens* chitinases ChiA and ChiB

The environment of a protein provides, at least in part, the relevant context necessary for its function. Therefore, the subcellular localization of a protein provides information about its biological function. Both putative *C*. *perfringens* chitinases, ChiA and ChiB, were predicted to be extracellular proteins by the CELLO subcellular localization predictor (S1 Table). Further analysis indicated that secretion of the chitinases ChiA and ChiB might occur through different pathways. SecretomeP predictions of the putative chitinases showed a SecP score > 0.8 for both putative chitinases, indicating possible secretion via a Sec-independent pathway (non-classical secretion). As SecretomeP can give high SecP scores to proteins containing a signal peptide, this information was combined with SignalP predictions to identify possible conventional signal peptides. For ChiA, a “standard" secretory signal peptide transported by the Sec translocon and cleaved by Signal Peptidase I (Sec/SPI) was identified with 91.1% probability (S1 Table). The cleavage site of the signal peptide was predicted to be located between AA34 and 35 (TKA-KE; 68.33% probability of the cleavage site prediction). No signal peptide was identified for ChiB, indicating that this enzyme is predicted to be secreted by a non-classical secretion mechanism.

The chitinase genes are located on the NELoc-1 pathogenicity locus, in close proximity to each other and having the same orientation. Only 85 nucleotides are located in between *chiA* and *chiB*, containing a potential ribosome binding site (Shine-Dalgarno sequence; S1 Fig). The expression of the chitinases genes on RNA level was assessed using qPCR. The presence of RNA of both chitinases was demonstrated when *C*. *perfringens* CP56 was grown in nutrient rich medium and mucus-supplemented medium, with the expression being the highest in the latter (S2 Fig).

### *In silico* physiochemical properties analysis

The alignment of the amino acid sequence of both chitinases using protein-protein BLAST identified 29.06% sequence identity (with 69% coverage) between both enzymes. This clearly indicates that the presence of both chitinases in the *C*. *perfringens* genome is not the result of a gene duplication event. The physicochemical properties of the putative chitinases ChiA and ChiB were predicted by the ExPASy ProtParam tool (S2 Table). The molecular weight of both chitinases was around 65–66 kDa. Both chitinases have a similar theoretical isoelectric point (pI) around 5, predicting them to be acidic in nature. Protein stability was assessed by focussing on the instability index, aliphatic index and the grand average of hydrophobicity (GRAVY) index. Both chitinases are classified as stable proteins (instability index below 40), with good thermostability (as indicated by a high aliphatic index, which is a measure for the thermostability of globular proteins). As a similar aliphatic index was obtained for both putative chitinases, similar temperature optima are expected. The obtained GRAVY index around -0.5 indicated that both chitinases are hydrophilic enzymes.

### ChiA and ChiB are members of the glycosyl hydrolase family 18

**Fig 1:**
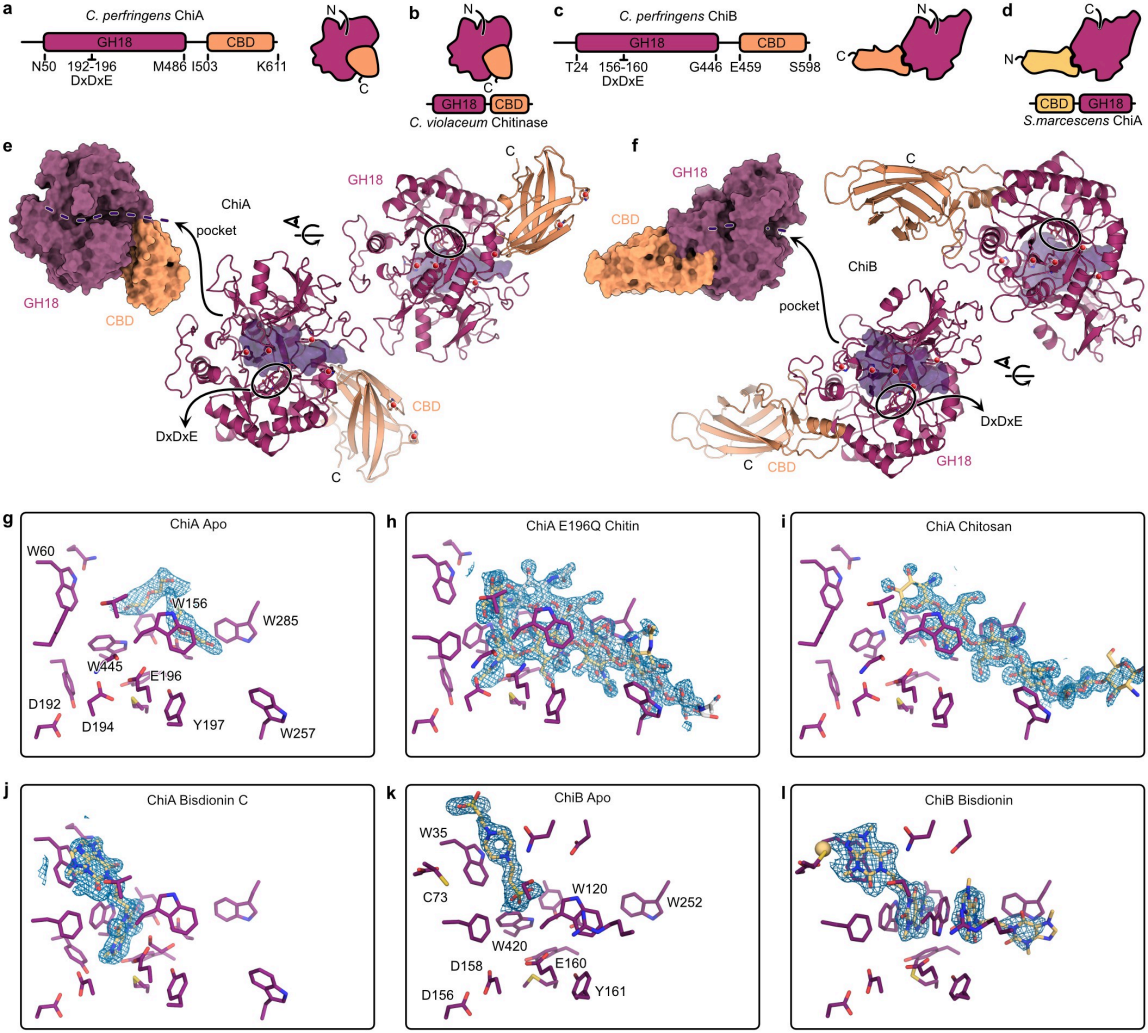
Crystal structures of chitinases ChiA and ChiB. (A, C) Schematic overview of protein domain organization and overall structures of ChiA and ChiB, both consisting of an N-terminal glycosyl hydrolase 18 (GH18) Trios-phosphate Isomerase (TIM) barrel followed by a putative carbohydrate binding domain (CBD). (B, D) Comparison of overall protein structures of ChiA and ChiB with those of previously studied chitinases (*Chromobacterium violaceum* and *Serratia marcescens*). (E,F) Surface representation of the protein crystal structures of ChiA and ChiB identifying the glycosyl hydrolase 18 domain (purple), carbohydrate binding domain (orange) and their open and tunnel shaped binding cleft, respectively, containing the DxDxE binding motif. The transparent purple volume in the cartoon representation of the crystal structures describes the ligand-binding pocket. (G,H, I, J, K, L) Ligand interactions of ChiA or ChiB with substrates: chitin, chitosan or bisdionin C. Ligand OMIT maps are shown as blue mesh representing the difference electron density Fourier coefficient mFo-DFc contoured at + 3 σ carved 2A around the ligands. Maps were generated by performing reciprocal space coordinate refinement after randomizing all atoms on average 0.4 Å in the absence of the ligands.

To gain a better understanding of the function of ChiA and ChiB we sought to obtain their structures. We succeeded in obtaining several crystal structures of recombinant ChiA and ChiB in apo (unbound) and ligand-bound forms to resolutions between 1.30 Å and 1.85 Å (S3 Table). Both ChiA and ChiB encode for an N-terminal glycosyl hydrolase family 18 (GH18) Trios-phosphate Isomerase (TIM) barrel followed by a C-terminal putative carbohydrate binding domain (CBD) (Fig 1A and 1C). This C-terminal CBD organization has previously been observed in a chitinase of *Chromobacterium violaceum* (PDB 4txg) (Figs 1B and S3). When comparing the overall structures of the chitinases, the unexpected location of the ChiB CBD becomes apparent. As compared to ChiA and the *Chromobacterium violaceum* chitinase, the CBD of ChiB is located on the opposite side of the TIM barrel. This CBD-location of ChiB is similar to the one occupied by the *Serratia marcescens* ChiA CBD. However, in *Serratia marcescens* this CBD is located at the N-terminus of the protein (Figs 1D and S3). For both chitinases, the CBD leads to a cleft in the GH18 TIM barrel domain lined with Trp residues which harbours at its base the canonical chitinase active site with a DxDxE motif. The cleft of ChiB is covered by a loop of the TIM barrel leaving only the sides open to the solvent (Fig 1E and 1F).

### Access and ligand interactions within the binding pocket

The accessibility of the CBD could impact ligand interaction and therefor the functionality of ChiA and ChiB. In the Apo form of ChiA, the binding pocket is occupied by somewhat disordered molecules originating from the crystallization condition (Fig 1G). The active site motif is trapped in the active conformation with D194 and E196 sidechains engaging in a low-barrier hydrogen bond. The E196Q point mutant of ChiA was generated to capture the ligand within a co-crystal structure. E196 is expected to be protonated in the catalytically competent state and act as a general acid/base to protonate the glycosidic bond during the substrate hydrolysis [28,29]. The chitin oligosaccharide (GlcNAc_6_) occupies two conformations (Fig 1H). In the first, bound conformation, the oligosaccharide has descended towards the active site occupying positions -2 to +3. The GlcNAc at position -1 is in the boat conformation, priming it for the formation of the oxazolinium reaction intermediate. In the second, encounter conformation, the elongated oligosaccharide is located at the surface of the cleft and only engages the CBM side of the cleft. This second conformation resembles the chitosan oligosaccharide (GlcN) bound conformation (Fig 1I). The missing acetyl group is required for the formation of the oxazolinium reaction intermediate and can consequently not be hydrolyzed. The -2 and -1 positions, where the product disaccharide is bound, are also the location where the inhibitor Bisdionin C binds (Fig 1J). The inhibitor molecule forces D194 into its resting conformation where it engages D192 in a low-barrier hydrogen bond as the other conformation would cause steric clashes. A second low occupancy or highly disordered Bisdionin C molecule might be bound in the vicinity of W257, more towards the CBD. Overall ChiA displays the hallmarks of a chitinase processing chitin from its non-reducing end towards the reducing end.

As in ChiA, the sizeable substrate binding pocket of Apo ChiB contained a PIPES buffer molecule originating from the crystallization condition (Fig 1K). The makeup of the binding pocket is conserved, except for the longer loop closing of the cleft and the entry of the cleft near the ChiB CBD. Neither differences should prohibit the binding of a linear chitin oligosaccharide. The active site motif is trapped in the resting conformation with a low-barrier hydrogen bond between D156 and D158. Attempts at obtaining substrate-bound structures failed but the substrate binding mode can be somewhat deduced from the structure of ChiB in complex with inhibitor Bisdionin C (Fig 1L). Two Bisdionin C molecules are observed in the ChiB binding site. The first molecule binds at nearly the same position as in ChiA but shifted approximately 2 Å towards the surface of the cleft. This position enables the Bisdionin C molecule to participate in the coordination of a divalent cation. The cation is part of the crystallization solution and is not present in the Apo structure. The second Bisdionin C molecule occupies the other half of the binding pocket. Each inhibitor molecule engages a side of the Trp120 indole ring with extensive van der Waals contacts which switched changes rotamer from t-105 to m0 causing it to project itself into the binding cleft. Both inhibitor molecules together occupy the ligand binding site from position -2 to +3. Overall, ChiB displays the hallmarks of a chitinase processing chitin from its reducing end towards the non-reducing end.

### Glycosyl hydrolase family 18 enzymes are associated with pathogenic *C*. *perfringens* type G strains

Possible functional redundancy of the chitinases in the *C*. *perfringens* genome was assessed by searching both the CAZy database (www.cazy.org) and the NCBI database for the presence of other *C*. *perfringens* enzymes belonging to glycosyl hydrolase families 18 (GH18) or 19 (GH19), which both contain chitinases. No GH19 family members were found in *C*. *perfringens*, whereas 22.2% (4/18) of the genomes in the CAZy database and 7.2% (56/778) of the *C*. *perfringens* genomes in the NCBI dataset contained GH18 containing proteins. The large majority of these GH18 domain containing proteins were found on the *netB-*plasmid of *C*. *perfringens* type G strains (100% (4/4, CAZy database) or 85.7% (48/56, NCBI dataset) of the *C*. *perfringens* strains with GH18 also contained NetB). In 8 *netB*-negative *C*. *perfringens* strains from the NCBI dataset GH18 family proteins were identified. These strains were isolated from pigs (n = 2), goats (n = 1), manure treated soil from a research farm (n = 1) or chickens (n = 4) in Australia, Belgium, China or Canada. In three of these chicken isolates, both the ChiA and the ChiB protein were identified, whereas the GH18 containing proteins from the other isolates showed only limited identity with ChiA or ChiB (S4 Table). To confirm the finding that the ChiA and ChiB isolates are solely found in chicken isolates and are linked to the *netB-*plasmid of type G strains, a diverse collection of *C*. *perfringens* strains, obtained from different hosts and geographical locations (Belgium, Denmark,…), was screened using PCR to assess the prevalence of *chiA* and *chiB*. All pathogenic *netB*-positive type G strains isolated from broilers (30/30) tested positive for the presence of both genes. In contrast, the genes *chiA* and *chiB* were not present in the commensal *netB*-negative type A strains isolated from broilers (0/48). In addition, the strains isolated from non-broiler hosts all tested negative: layer (0/7), cattle (enterotoxaemia 0/6; healthy calves 0/8, ruminating cattle 0/3), sheep (0/7), horse (0/5), dog (0/2), pig (0/1), human (gangrene 0/1), deer (0/1) and goat (0/3) (S5 Table).

### ChiA and ChiB show preferential substrate binding

**Fig 2:**
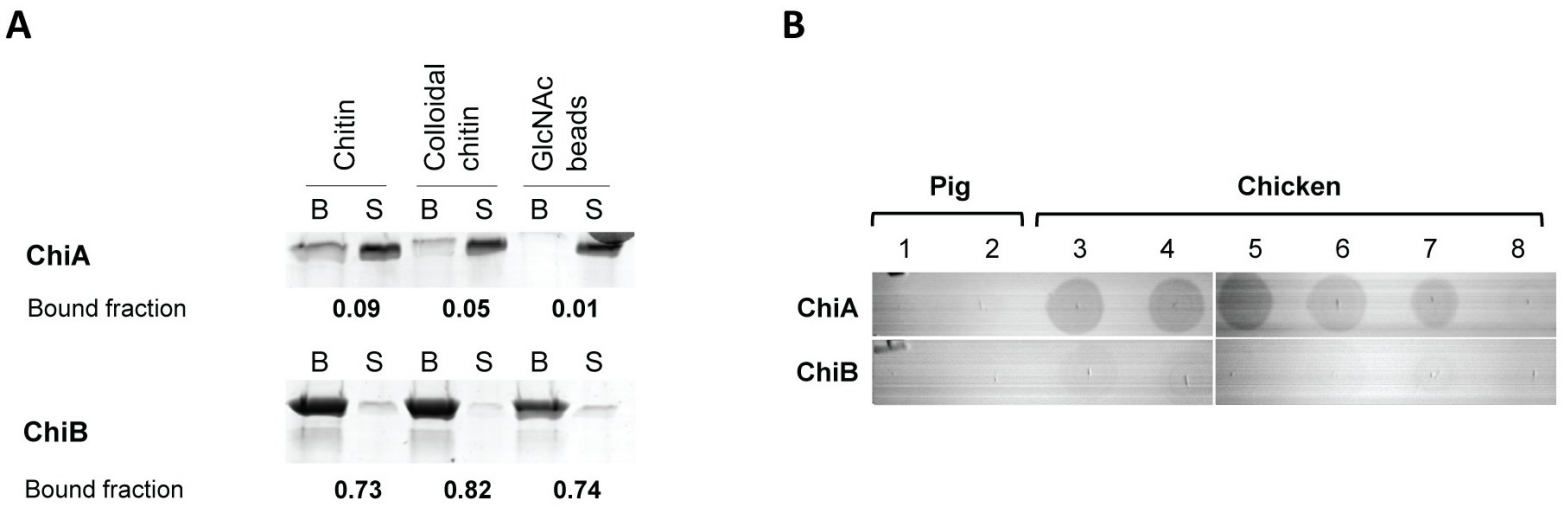
Binding of the inactive chitinases ChiA or ChiB towards (A) crystalline chitin, colloidal chitin or GlcNAc-covered beads or (B) crude chicken intestinal mucus. (A) In solution binding assay: The amount of bound and unbound protein to the substrate was visualised using SDS-PAGE and subsequent Coomassie staining. B = bound fraction, S = supernatants representing the unbound protein fraction. Intensity of the bands was quantified as OD/mm^2^. The bound protein fraction was calculated by dividing the intensity of bound protein by total protein for each substrate. Assay was performed twice, one representative figure is shown. (B) Dotblot: Either porcine mucus type II (1), porcine mucus type III (2) or crude chicken mucus (3–8; 6 biological replicates) were spotted onto the membrane. Membranes were incubated with either recombinant ChiA or ChiB. The amount of bound enzyme was visualized using anti-HIS-antibody. Assay was performed twice, one representative figure is shown.

Substrate binding is a prerequisite for an enzyme to exert its enzymatic activity. Characterisation of the binding properties of enzymes aids the identification of their potential substrates. To examine whether the *C*. *perfringens* chitinases interact with various polysaccharides, a binding assay between the recombinant chitinases and either crystalline chitin, colloidal chitin or GlcNAc-coated beads was performed. The amount of bound and unbound protein to the substrate was visualised after which the fraction of bound protein was calculated (Fig 2A). For this assay, ChiB was able to bind to crystalline chitin (73%), colloidal chitin (82%) and GlcNAc-coated beads (74%), whereas ChiA showed only limited binding to the substrates (9%, 5% or 1% to respectively crystalline chitin, colloidal chitin or GlcNAc). As *C*. *perfringens* type G strains are intestinal pathogens, and chitin is not present in the intestinal mucosa, chitin is probably not the primary substrate of the putative chitinases *in vivo*. As both putative chitinases were classified as O-glycosyl hydrolases, they might exert their action on mucin-type O-glycans in the intestinal mucus. The ability of the recombinant enzymes to interact with crude chicken intestinal mucus or porcine mucus was assessed using a dot blot assay (Fig 2B). None of the enzymes were able to bind to porcine type II or III mucus. No binding of ChiB could be detected to the substrates. This contrasts with ChiA, where no binding was observed towards either of the porcine mucins, but a clear interaction of ChiA with the crude chicken mucus was observed (5 out of 6 biological replicates of chicken mucus). These findings indicate that both chitinases have different substrate preferences: ChiB has a higher affinity towards chitin whereas ChiA binds to small intestinal chicken mucus.

### Activity of chitinases towards colloidal chitin and 4-MU-(GlcNAc)_1-3_ substrates

The putative chitinases belong to the glycosyl hydrolases family 18, a family that harbours both active chitinases that hydrolyse glycosidic bonds as well as chitinase-like proteins which only bind to but do not cleave the substrate. To assess whether the *chiA* and *chiB* genes encode enzymatically active proteins, both putative chitinases were recombinantly expressed and their enzymatic activity towards colloidal chitin was characterized (Table 1). Both chitinases demonstrated activity towards colloidal chitin. However, a higher hydrolysis rate and higher catalytic efficiency (*k*_cat_/*K*_M_) was observed for chitinase ChiB as compared to ChiA. To elucidate whether the observed enzymatic activity towards chitin was caused by exo- or endo-glycosidase activity (cleaving either terminal or internal glycosidic linkages in the polymer, respectively) of the chitinases, further assays using the fluorescently labelled chitin pseudo-substrates 4-MU-(GlcNAc)_1-3_ were performed. Both chitinases were inactive towards 4-MU-GlcNAc, whereas both 4-MU-GlcNAc_2_ and 4-MU-GlcNAc_3_ were suitable substrates (Table 1). This indicates that both chitinases function as endo-chitinases. Overall, a higher hydrolysis rate and higher catalytic efficiency (*k*_cat_/*K*_M_) was observed for chitinase ChiB as compared to ChiA. Due to unambiguous model fitting, no kinetic parameters could be determined for ChiB using 4-MU-(GlcNAc)_3_ as a substrate.

**Table 1:** Kinetic parameters of the fitted models to the enzymatic activity data of ChiA or ChiB towards the pseudo-chitin substrates 4-MU-GlcNAc_2_, 4-MU-GlcNAc_3_ or colloidal chitin. K_cat_ = catalytic constant, K_m_ = substrate concentration given that the reaction rate reaches ½ V_max_, K_cat_/K_m_ = Specificity constant, ND: kinetic parameters not determined due to unambiguous model fitting for ChiB using 4-MU-(GlcNAc)_3_. BDL: “Below detection limit”.

	ChiA			ChiB		
	*K*_cat_ (s^-1^)	*K*_m_ (μM)	*K*_cat_/*K*_m_ (s^-1^ μM^-1^)	*K*_cat_ (s^-1^)	*K*_m_ (μM)	*K*_cat_/*K*_m_ (s^-1^ μM^-1^)
**GlcNAc**	BDL	BDL	BDL	BDL	BDL	BDL
**GlcNAc**_**2**_	3.09	145.8 ± 91.3	0.021	15.2	24.3 ± 9.7*10^−3^	0.627
**GlcNAc**_**3**_	0.0993	2.6 ± 2.3*10^−4^	0.039	ND	ND	ND
**Chitin**	0.176	14.8 ± 6.2	0.012	0.263	6.6 ± 1.2	0.040

### Effect of temperature and pH on enzymatic activity towards pseudo-chitin substrates

**Fig 3:**
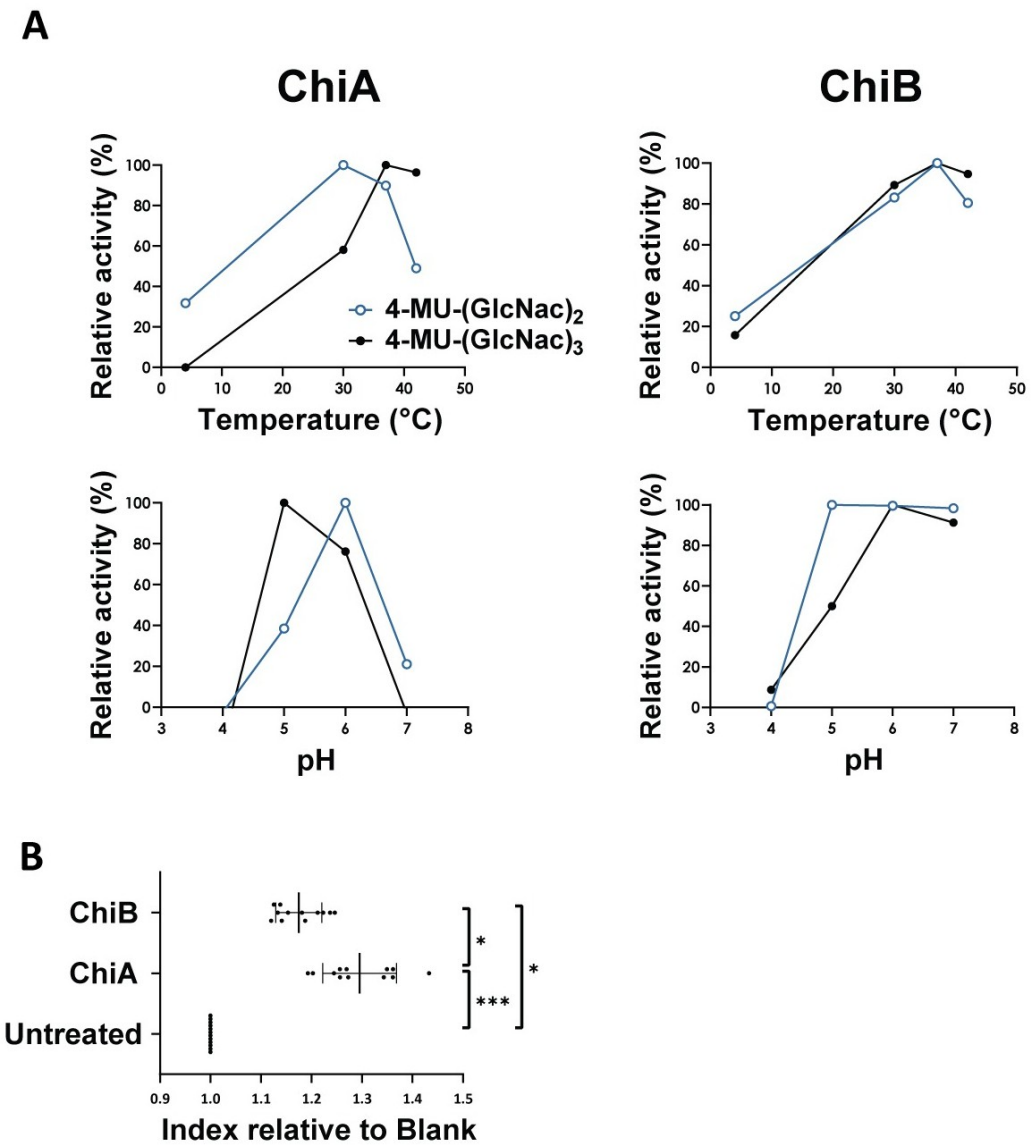
Enzymatic activity of chitinases ChiA and ChiB towards (A) pseudo-chitin substrates (effect temperature and pH) or (B) crude chicken mucus. (A) Effect of temperature and pH on chitinase activity towards pseudo-chitin substrates (4-MU-GlcNAc_2_ and 4-MU-GlcNAc_3_). The enzymatic activity of ChiA (left) or ChiB (right) at different temperatures was assessed at a constant pH of 5 (upper panels), whereas the effect of the pH was monitored at a constant temperature of 42°C (lower panels) (no replicates). (B) Chitinase activity towards chicken mucus using turbidity assay. Biological replicates of crude intestinal chicken mucus (150 μg) were incubated with either recombinant ChiA, ChiB (15 μg) or PBS. After a one hour incubation period at 37°C, the turbidity of the mixture was measured at an OD-value of 450 nm. The relative index was calculated by dividing the OD of the chitinase-treated mucus by the OD of the untreated mucus sample for each mucus sample. Lines indicate the means with their respective standard deviations. Significant differences are indicated with ‘*’ (p≤0.05). ‘**’ (p≤0.01) and ‘***’ (p≤0.001).

Since it is hypothesized that the putative chitinases play a role during *C*. *perfringens* pathogenesis, their ability to be enzymatically active at temperatures and acidity levels representative of the gastrointestinal tract was assessed. The effect of temperature and pH on the enzymatic activity of both enzymes towards 4-MU-GlcNAc_2_ and 4-MU-GlcNAc_3_ was analysed (Fig 3A). Temperature optima (at a constant pH5) were found at 37°C, except for ChiA cleaving 4-MU-GlcNAc_2_ where maximal hydrolysis rate was reached at 30°C. Lower temperatures reduced the activity of the recombinant chitinases, although most often not completely when reaching 4°C. In addition, temperatures exceeding the optimal temperature, reduced the enzymatic activity. However, at 42°C (chicken body temperature), still high activity was measured. Chitinase activity (for both ChiA and ChiB) was highest at a pH between 5 and 6 (at a constant 42°C). An excessively acidic or alkaline environment inhibited ChiA enzymatic activity. The enzymatic activity of ChiB was only hampered when the pH was too low. Both enzymes were highly active at 42°C and pH 5–6, the biologically relevant conditions inside the gastrointestinal tract of broiler chickens.

### Intestinal mucus, a chitinase substrate of biological significance

Since chitinase ChiA was able to bind to chicken intestinal mucus, the enzymatic activity of the chitinases towards crude chicken mucus was assessed using a turbidity assay (Fig 3B). With the addition of either chitinase, the turbidity of the mucus-containing mixture increased, indicating mucosal breakdown. The factor to which extent the OD-value increased was calculated relative to the blank conditions. The turbidity index of mucus treated with ChiA or ChiB was significantly different as compared to untreated mucus (p≤0.0001 for ChiA; p = 0.0429 for ChiB). The activity of ChiA on crude chicken mucus was higher as compared to ChiB (p = 0.0429), indicating a higher preference of the ChiA towards chicken mucus.

**Fig 4:**
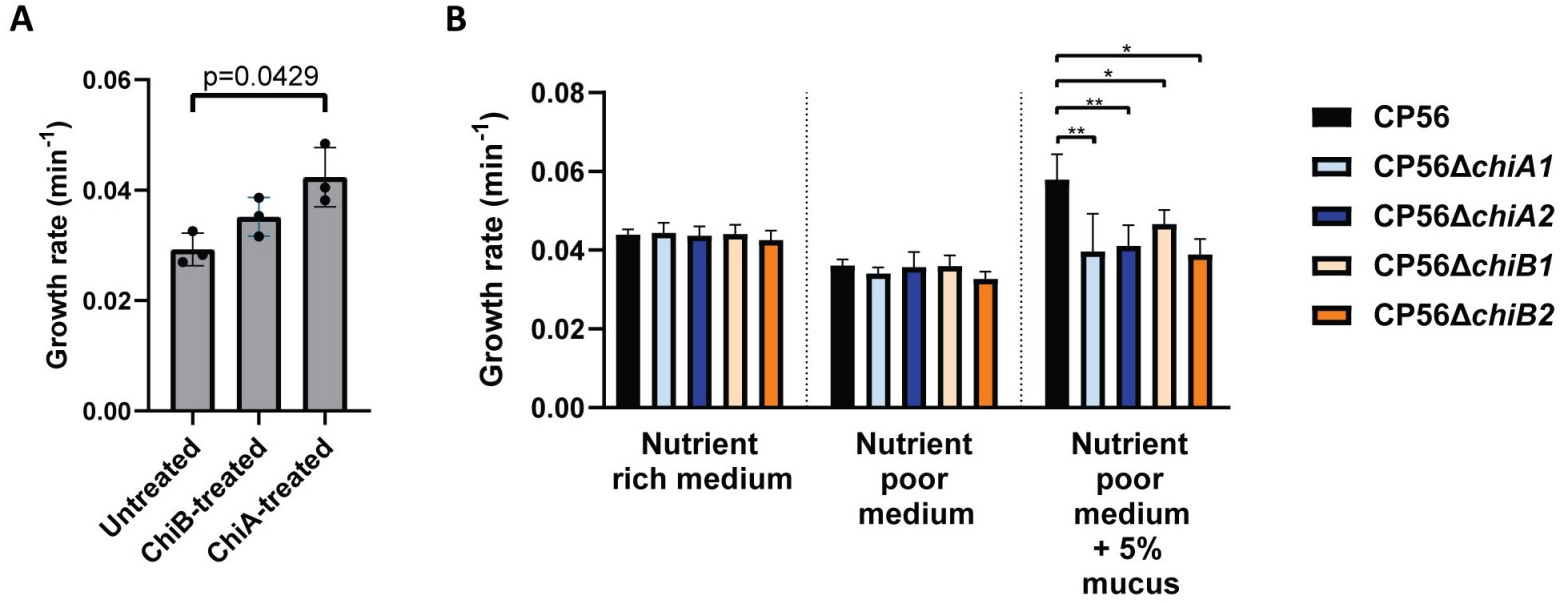
Effect of mucus on the growth of *C*. *perfringens*. (A) Growth analysis of *C*. *perfringens* CP56 in media supplemented with 5% chicken intestinal mucus pre-treated with either PBS (= untreated), 15μg of chitinase ChiA or ChiB for one hour. Growth rate is defined as the slope during exponential rate. Bars indicate the means with their respective standard deviations. (B) Growth analysis of wild-type and chitinase mutant strains in different media. Wild type CP56 (Black), CP56Δ*chiA1* (light blue), CP56Δ*chiA2* (dark blue), CP56Δ*chiB1* (light orange) or CP56Δ*chiB2* (dark orange) were grown in either nutrient rich medium, nutrient poor medium or nutrient poor medium supplemented with 5% crude chicken mucus. Growth rate is defined as the slope during the exponential phase. Bars indicate the means with their respective standard deviations. Significant differences are indicated with ‘*’ (p≤0.05). ‘**’ (p≤0.01). ‘***’ (p≤0.001) and ‘****’ (p≤0.0001).

The hypothesis that chitinases aid the breakdown of intestinal mucus to facilitate bacterial growth was validated through the treatment of chicken intestinal mucus with either ChiA, ChiB or PBS (as a negative control). The growth rate of wild type CP56 was assessed in media supplemented with either chitinase- or PBS-treated mucus. The growth rate of the bacteria grown in media supplemented with 5% ChiA-treated mucus increased significantly as compared to the untreated mucus (p = 0.0429) (Fig 4A), indicating that chitinases indeed aid nutrient acquisition of the pathogen to some extent. This effect was lower and not significant when supplementing the medium with ChiB-treated mucus (p = 0.6620).

### Generation of *C*. *perfringens* ChiA and ChiB mutants

To further address the role of *C*. *perfringens* chitinases ChiA and ChiB, mutant strains from each of the chitinase genes (*chiA* or *chiB*) were constructed from the pathogenic *C*. *perfringens* type G strain CP56, using the ClosTron mutagenesis system. PCR using a forward primer targeting the ClosTron insert and a reverse primer downstream of the insertion site showed correct insertion of the ClosTron insert in either the *chiA* or *chiB* gene. No additional ClosTron insertions were detected using dPCR [30].

Despite multiple attempts, chitinase mutant strains could not be complemented. Large plasmid uptake severely hampered the phenotype of the strains, thereby significantly reducing growth properties. Alternatively, whole genome sequencing was performed to assess potential secondary mutations of the mutant strains (two for each chitinase: CP56Δ*chiA1*, CP56Δ*chiA2*, CP56Δ*chiB1* and CP56Δ*chiB2*) that could affect the virulence phenotype and consequently hamper the outcome of subsequent experiments.

As compared to the CP56 wild-type genome, no INDELs were found in all mutant strains. A total of seven, five, two and eight SNPs were identified in the genomes from respectively CP56Δ*chiA1*, CP56Δ*chiA2*, CP56Δ*chiB1* and CP56Δ*chiB2*. In addition, these limited number of SNPs were not present in more than one strain. An overview of the identified gene products and their predicted function is given in S6 and S7 Tables. The SNPs with a potential impact on mucin degradation, both its carbohydrate and peptide moiety, will be highlighted here [31]. Mutant CP56Δ*chiA1* harbours a missense-variant in a hypothetical protein containing a DUF1667 domain. The function of this domain remains unknown, however in a small amount of cases it has been found in archaeal and bacterial hypothetical proteins, some of which have been annotated as potential metal-binding proteins, often oxidoreductases and dehydrogenases. Two cases have been described in literature in which a DUF1667 region was located in glycerol-3-phosphate dehydrogenases, enzymes with a role in lipid metabolism [32,33]. Mutant strain CP56Δ*chiA2* harbours six missense variants and one SNP that resulted in a stop codon. One of these SNPs is located in a hypothetical protein that has 97.78% sequence identity with the Zinc-dependant exopeptidase M28, a bacterial leucyl aminopeptidase that can potentially lead to protein degradation of the protein backbone of mucin. The two SNPs in mutant CP56Δ*chiB1* are not located in genes with a potential mucin-degrading function. CP56Δ*chiB2* harbours a SNP in a DUF4091 domain-containing protein. Although the function of this domain is uncharacterised, it is often conserved in N-acetylgalactosaminidases. These types of enzymes are known to catalyse the hydrolysis of the O-glycosidic bond between GalNAc at the reducing end of a mucin sugar chain and serine/threonine of the proteins [31,34,35].

Except from the ClosTron insertion in the *chiA* or *chiB* gene (for respectively CP56Δ*chiA1/2* or CP56Δ*chiB1/2*), no further variants were detected in the well-known toxin genes *cpa* or *netB*, either of the pathogenicity loci (NELoc-1, NELoc-2 or NELoc-3) nor the genes encoding the VirSR regulatory system which is known to control the expression of virulence genes.

We sought further support for these findings by assessing the NetB producing capacity of the mutant strains. A haemolytic assay was performed in which no difference in NetB activity of the different culture supernatant was quantified (S4 Fig), indicating no impact of the SNPs on the main toxin associated to virulence. In addition, the growth properties of the mutant strains were evaluated to determine a potential overall effect of the detected SNPs. Wild-type and mutant strains had an equal growth rate at mid-exponential phase (CP56: 0.0439 ± 0.0014 min^-1^; CP56Δ*chiA1*: 0.0444 ± 0.0026 min^-1^; CP56Δ*chiA2*: 0.0437 ± 0.0023 min^-1^; CP56Δ*chiB1*: 0.0441 ± 0.0023 min^-1^; CP56Δ*chiB2*: 0.0425 ± 0.0024 min^-1^) (Figs 4B and S5).

Taken together, the genetic analysis of the mutant strains (only limited effect of SNPs that are not located in virulence-related regions), their equal NetB activity and their equal growth compared to the wild-type strain gave us confidence that possible differences in phenotype between the strains are unlikely to be the result from the secondary mutations.

### *C*. *perfringens* chitinase mutant strains show attenuated growth in chicken mucus

To further investigate the biological significance of the *C*. *perfringens* chitinases, their importance during growth of *C*. *perfringens* in mucus-containing media was assessed using either a wild-type strain or its isogenic mutants which lack either of the chitinase genes. Growth analysis was performed for the wild-type or chitinase mutant strains (CP56Δ*chiA1*, CP56Δ*chiA2*, CP56Δ*chiB1* or CP56Δ*chiB2*) in different media (Figs 4B and S5). When grown in nutrient rich or nutrient poor medium, no difference in growth between the wild-type and either of the chitinase mutant strains could be observed (p = 0.3964 and p = 0.2619, respectively). The wild-type strain grew faster in nutrient poor medium supplemented with 5% crude chicken mucus as compared to non-supplemented nutrient poor medium (p = 0.0006), indicating that crude mucus is a good nutrient source. In mucus-supplemented medium all mutant strains grew slower as compared to the wild-type strain (p = 0.0046 for CP56 vs. CP56Δ*chiA1*; p = 0.0013 for CP56 vs. CP56Δ*chiA2;* p = 0.0297 for CP56 vs. CP56Δ*chiB1;* p = 0.0172 for CP56 vs. CP56Δ*chiB2*), indicating that the chitinases are advantageous in the utilisation of mucus as an additional growth substrate.

**Fig 5:**
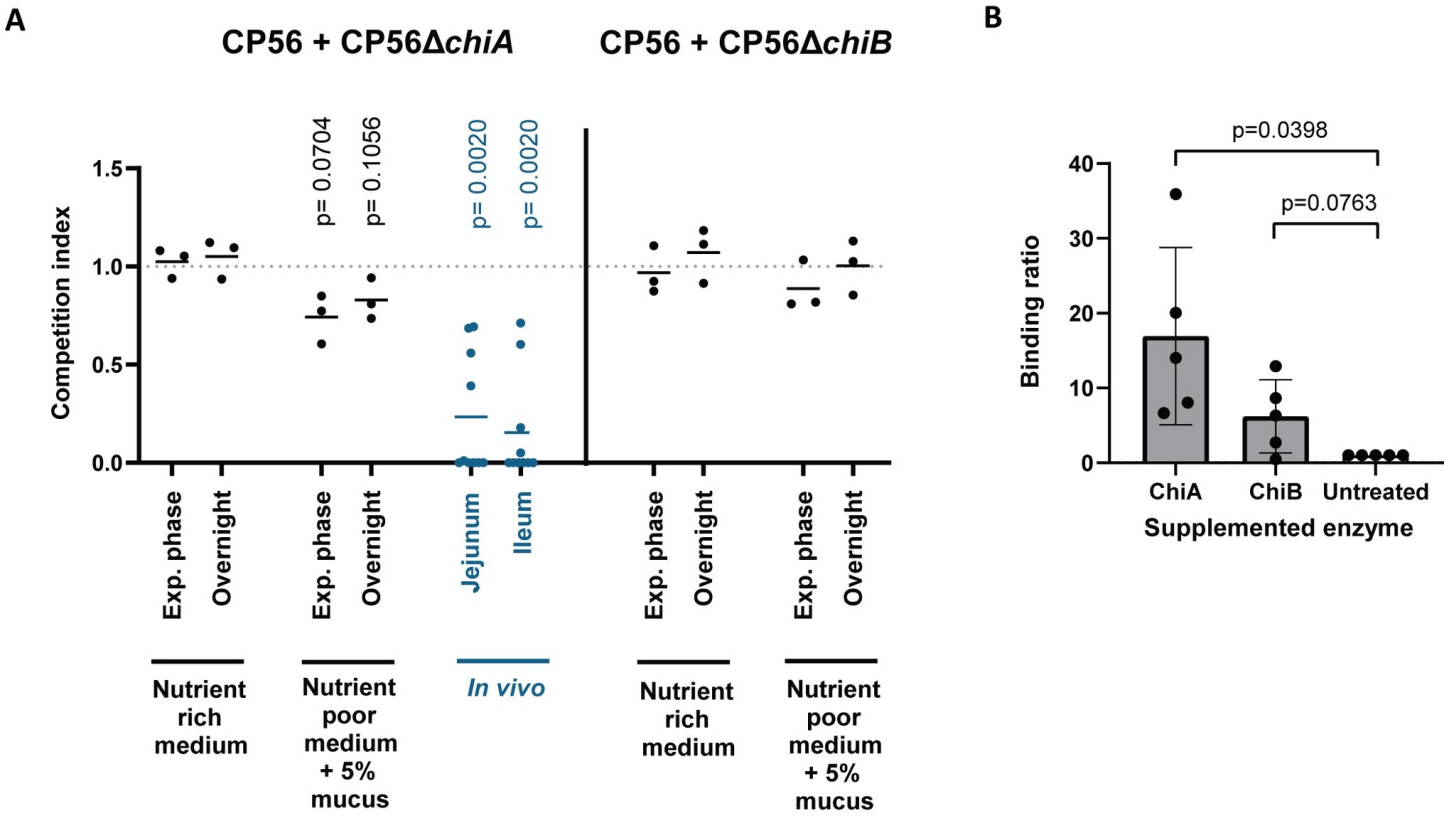
Effect of mucus on colonisation *C*. *perfringens*. (A) Competition assay of *C*. *perfringens* wild-type strain CP56 and mutant strains CP56Δ*chiA1* or CP56Δ*chiB1* in either *in vitro* or *in vivo* growth conditions. *In vitro*: An equal mix of wild-type and mutant strain was grown in either nutrient rich medium or nutrient poor medium supplemented with 5% chicken intestinal mucus. Samples were taken at the exponential growth phase and at saturation after overnight incubation. *In vivo*: 18-days old broiler chickens were inoculated with an equal mix of wild-type and CP56Δ*chiA1* mutant strain. After 24 hours, samples were taken from the intestinal content (jejunum or ileum). The amount of wild-type or mutant strain in the samples was determined using dPCR. The competition index is defined as the ratio of the mutant on wild-type strain, divided by the respective ratio in the inoculum. Lines indicate the means. (B) Mucus binding assay of wild-type CP56 *C*. *perfringens* strain in media supplemented with recombinant chitinases, either ChiA or ChiB. Washed *C*. *perfringens* overnight culture was added to the wells containing a mucus agar layer, supplemented with either 50 μg of recombinant enzyme (ChiA or ChiB) or an equal volume of PBS as a negative control. Wells were anaerobically incubated for 90 min at 37°C after which the bound bacteria were quantified through titration. The binding ratio is defined as the percentage of bacteria bound to the mucus in supplemented media as compared to non-supplemented conditions. Bars indicate the means with their respective standard deviations.

To further strengthen these findings, bacterial competition assays between the wild-type and one of either of the chitinase mutant strains in mucus-containing media were performed. Therefore, different media were inoculated with an equal amount of wild-type and mutant strain, either CP56Δ*chiA1* or CP56Δ*chiB1*. For both mixtures (CP56 with CP56Δc*hiA1* or CP56 with CP56Δ*chiB1*), the competition index (ratio mutant:wild type) in nutrient rich medium did not differ from 1 (both during the exponential phase and at saturation), indicating that both strains were growing at an equal rate in nutrient rich medium (Exponential phase: ChiA p = 0.6353 and ChiB p = 0.6917; Overnight: ChiA p = 0.4741 and ChiB p = 4724) (Fig 5A). When mucus is the main nutrient source in the medium (i.e. mucus-supplemented nutrient poor medium), no significant drop in competition index could be observed when growing CP56 together with CP56Δ*chiA1* (Exponential phase: p = 0.0704; Overnight p = 0.1056) or CP56Δ*chiB1* (Exponential phase: p = 0.2627; Overnight p = 0.9723).

### Role of chitinases during *C*. *perfringens* colonization

The digestion of mucus by chitinases may alter its composition, which could facilitate bacterial binding and may be a critical step during the initial colonization of the small intestine. To assess whether the chitinases could affect *C*. *perfringens* adherence to intestinal mucus, a mucus binding assay was performed. Addition of ChiA to CP56 increased the bacterial binding capacity to intestinal mucus as compared to non-supplemented conditions (Fig 5B, p = 0.0398). The effect of ChiB on the binding of CP56 to chicken mucus could not be statistically proven (p = 0.0763).

Since ChiA has more affinity towards mucus, the following *in vivo* studies were preformed using only the CP56Δ*chiA1* mutant strain. To study the importance of this chitinase during colonization, an *in vivo* colonization assay was performed in which 18-day old broilers were orally inoculated with a 50/50 mixture of wild-type and CP56Δ*chiA1* mutant strain. As a control, the inoculum was grown in an *in vitro* setting in different media, verifying that both strains were growing at an equal rate (Fig 5A). After oral administration to broilers, the mean competition index of the retrieved intestinal content samples significantly dropped both in the jejunum and ileum (Fig 5A; p = 0.0020 for both), indicating that more wild-type strain was present in the intestinal content as compared to mutant.

### Chitinases are involved in the pathogenesis of necrotic enteritis

**Fig 6:**
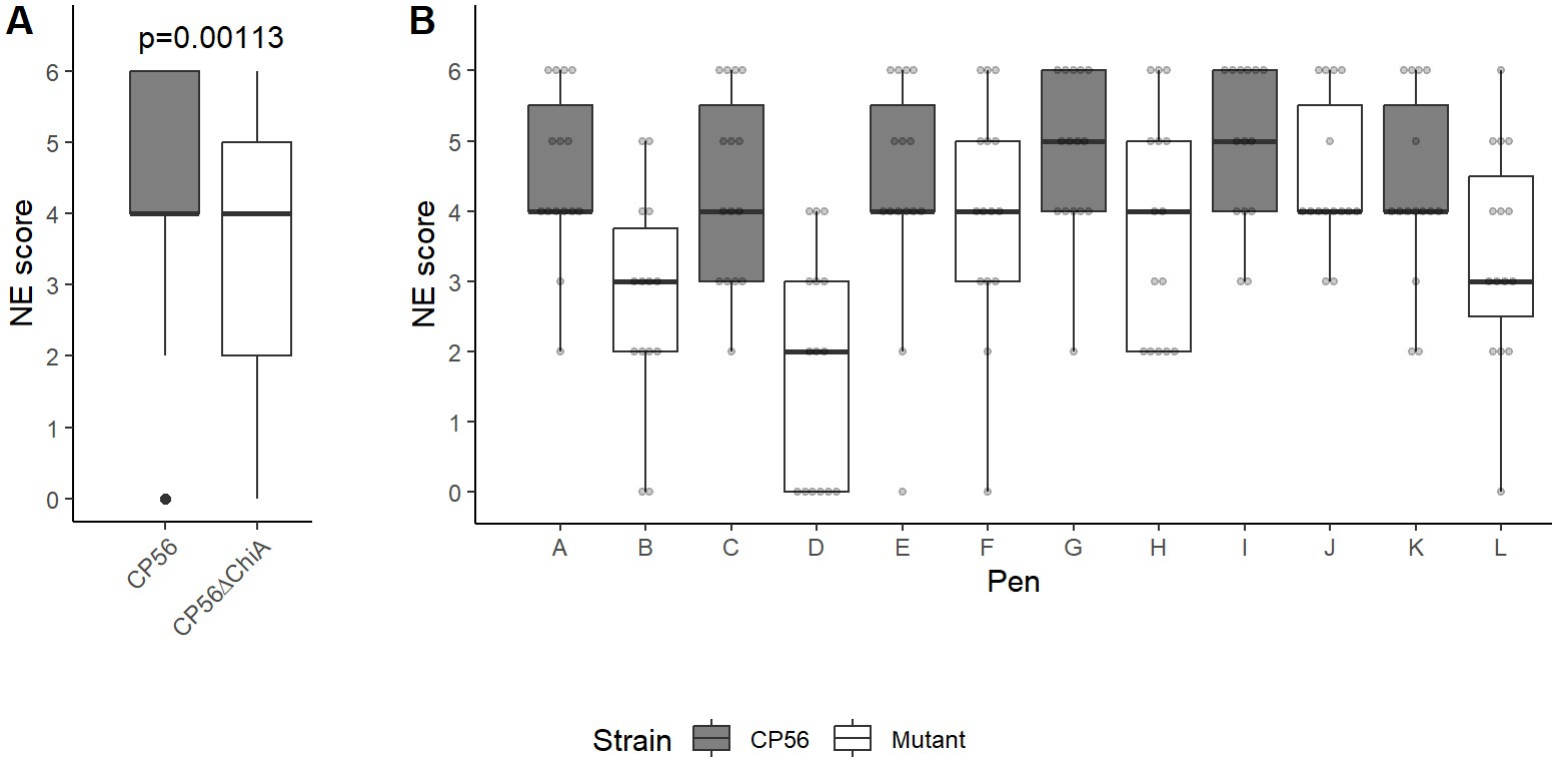
Necrotic enteritis *in vivo* trial. Predisposed broilers (six replicate pens per *C*. *perfringens* strain, 15 birds/pen) were challenged with *C*. *perfringens* (either wild type CP56 or mutant CP56Δ*chiA1*) on days 18 and 19 after which the severity of necrotic lesions was determined on day 20; score 0: no gross lesions; score 2: focal necrosis and ulceration (1–5 foci); score 3: focal necrosis and ulceration (6–15 foci); score 4: focal necrosis and ulceration (16 or more foci); score 5: patches of necrosis 2 to 3 cm long and score 6: diffuse necrosis. Overall boxplots per *C*. *perfringens* strain (A) are shown, as well as individual boxplots per pen (B). The grey dots indicate individual scores per bird. Statistical difference in disease severity was assessed using a cumulative link mixed model, with disease severity score (ordinal factor 0–6) as the response variable and *C. perfringens* strain (CP56 or CP56Δ*chiA1*) as a predictor variable, thereby accounting for non-independence of birds housed within the same pen by specifying “pen” as a random effect.

An *in vivo* NE trial was conducted to assess the biological significance of ChiA during the pathogenesis of *C*. *perfringens* infection. All broilers were predisposed to the disease by coccidiosis infection and fish meal supplementation, after which they were challenged with either the wild type CP56 or mutant CP56Δ*chiA1* strain. The mutant strain had a reduced ability to induce NE. Indeed, less animals suffered from severe lesions (scores 5–6; CP56Δ*chiA1*: 25%, CP56: 49%; p = 0.0128) while birds without lesions (score 0; CP56Δ*chiA1*: 12%, CP56: 1%; p = 0.1133) and more mild cases (scores 2–3; CP56Δ*chiA1*: 38%, CP56: 16%; p = 0.0170) were more prominent in the groups challenged with the mutant strain. In addition, the average lesion score of the birds challenged with the mutant strain (3.37 ± 1.72) was significantly lower as compared to birds challenged with the wild-type stain (4.52 ± 1.29) (p = 0.00113) (Fig 6).

## Discussion

Chitinases are produced by a wide variety of living organisms including bacteria, fungi, plants, insects, crustaceans, and mammals [36]. The main function of these glycosyl hydrolases is the degradation of chitin, the second most abundant biopolymer in the environment, to low molecular weight oligomers. Bacterial chitinases have been shown to be important in nutrient acquisition as well as environmental survival [37,38]. In addition, some chitinases expressed by pathogenic bacteria have been identified as crucial virulence factors in respiratory, gastrointestinal or urinary infections. Examples are chitinases expressed by *Listeria monocytogenesis*, *Legionella pneumophila* and *Salmonella* Typhimurium [39–41]. This research focussed on two putative chitinases (ChiA and ChiB) which are expressed by *C*. *perfringens* type G strains, the causative agent of NE in broilers [24]. To date, many factors regarding NE pathogenesis, including its early stages, remain elusive. Interestingly, the expression of both chitinase genes was previously shown to be upregulated during the early stages of pathogenesis, suggesting their importance during colonization [27]. Pathogenic *C*. *perfringens* is known to reside near the gastrointestinal epithelium, near the mucus layer [42]. This raises the question whether *C*. *perfringens* chitinases might be involved in the initial colonization through their interaction with the mucus layer.

Both chitinases were identified as enzymes belonging to the glycosyl hydrolase (GH) family 18, like most other chitinases from bacterial pathogens. These chitinases were predominantly found in *C*. *perfringens* type G strains, without any homologs belonging to GH18 or GH19 family members in *C*. *perfringens* strains isolated from other hosts, indicating the absence of functional redundancy in *C*. *perfringens* strains that cause intestinal disorders in non-avian hosts. Enzymes need to be enzymatically active to play a crucial role during pathogenesis. Indeed, both enzymes were enzymatically active towards multiple substrates such as colloidal chitin and pseudo-chitin substrates 4-MU-(GlcNAc)_2-3_. Even at a temperature of 42°C and pH levels around 5, enzymatic activity was present, indicating that *C*. *perfringens* chitinases can be active inside the gastrointestinal tract of chickens. The catalytic efficiencies towards colloidal chitin and 4-MU-(GlcNAc)_2-3_ of chitinase ChiB was higher as compared to ChiA and both are similar to or higher than the values of chitinases from other pathogenic bacteria (ChiA 0.021 μM^-1^s^-1^; ChiB 0.627 μM^-1^s^-1^ versus LmChiA 0.020 μM^-1^s^-1^ and LmChiB 0.190 μM^-1^s^-1^ from Listeria monocytogenes; BthChi74 0.010 μM^-1^s^-1^ from *Bacillus thuringiensis*; chitinase 0.000404 μM^-1^s^-1^ from *Pseudomonas aeruginosa* towards GlcNAc_2_) [41,43,44].

The substrate specificity greatly differs between both *C*. *perfringens* chitinases, with ChiB being more active towards chitin whereas ChiA prefers chicken intestinal mucus. Binding studies confirmed these findings. The preference of the enzymes towards either linear (chitin) or more branched substrates (mucus) is in accordance with the predicted substrate binding site (based on protein crystal structure) of both enzymes. Indeed, ChiB was predicted to have a typical substrate-binding cleft of subfamily A chitinases, suggested to facilitate the hydrolysis of oligosaccharide dimers from the polysaccharide chains such as chitin, by “sliding” the substrate through the substrate-binding cleft [45]. This contrasts with the wider catalytic cleft of chitinase ChiA, which could allow more branched substrates into its active site. Furthermore, the location of the additional unique loop identified near the active site of ChiA, suggests that it might serve as a flexible loop that undergoes a conformational change after binding of the substrate, thereby securing the substrate in the active site. Despite the lack of binding of ChiA to linear chitin (under our experimental conditions), some enzymatic activity could be detected. Potentially, only a weak binding between ChiA and chitin, without additional securing of the substrate by the loop, was present which was not able to withstand some high impact steps used in the binding protocol. A remark has to be made that process-related impurities generated during the production of the recombinant enzymes could affect the results, however chances are low. Combining the fact that the chitinases are unique to *C*. *perfringens* strains that were isolated from broilers suffering from NE and the preference of ChiA towards chicken intestinal mucus and not porcine mucins, which has a different mucus composition, can perhaps explain the host-specificity of the pathogenic strains towards broilers [46]. The composition of O-glycan moiety of avian mucins is known to be enriched in sulphated monosaccharides and N-acetyl-d-neuraminic acid, resulting in an overall negative charge of the mucins [47]. The interaction between *C*. *perfringens* chitinases, host mucus composition and potential mucus altering factors such as (but not limited to) feed composition, additional infections or host age, is an interesting field to explore [47,48].

Vertebrates, except for certain fish and amphibian species, do not have the ability to synthesize chitin [13,14]. Despite the lack of chitin in the intestinal tract, various enzymes and proteins which are annotated as chitinases and chitin-binding proteins have been linked to pathogenesis of enteric bacterial diseases [11], now also including the chitinases of *C*. *perfringens*. Potential targets, other than chitin, are the β-1,4-linkage in GlcNAc-containing glycolipids and glycoproteins, which are present in the intestinal mucus layer [11,15]. The discrepancy in substrate preference of the enzymes raises the question if the term “chitinases” should still be used. The authors of this manuscript strongly recommend to refer to these enzymes as “O-glycosyl hydrolases” in future research to ensure scientific correctness.

During this research chitinase mutant *C*. *perfringens* strains were constructed. Despite multiple attempts, chitinase mutant strains could not be complemented and therefor alternative approaches were used. During this research, a combination of assays using either mutant *C*. *perfringens* strains or recombinantly produced proteins were utilised to study the biological role of the chitinases, thereby strengthening research findings. For each chitinase protein, two independent mutant strains were selected, displaying identical phenotypes in respect to growth properties and NetB activity. Mutant strains were sequenced thereby revealing no INDELs and only a few SNPs, which were different in all strains, thereby limiting the chances that the SNPs cause identical phenotypes in both mutant strains. Only limited effect of the SNPs was to be expected. No further variants were detected in the well-known toxin genes *cpa* or *netB*, either of the pathogenicity loci (NELoc-1, NELoc-2 or NELoc-3) nor the genes encoding the VirSR regulatory system which is known to control the expression of virulence genes.

**Fig 7:**
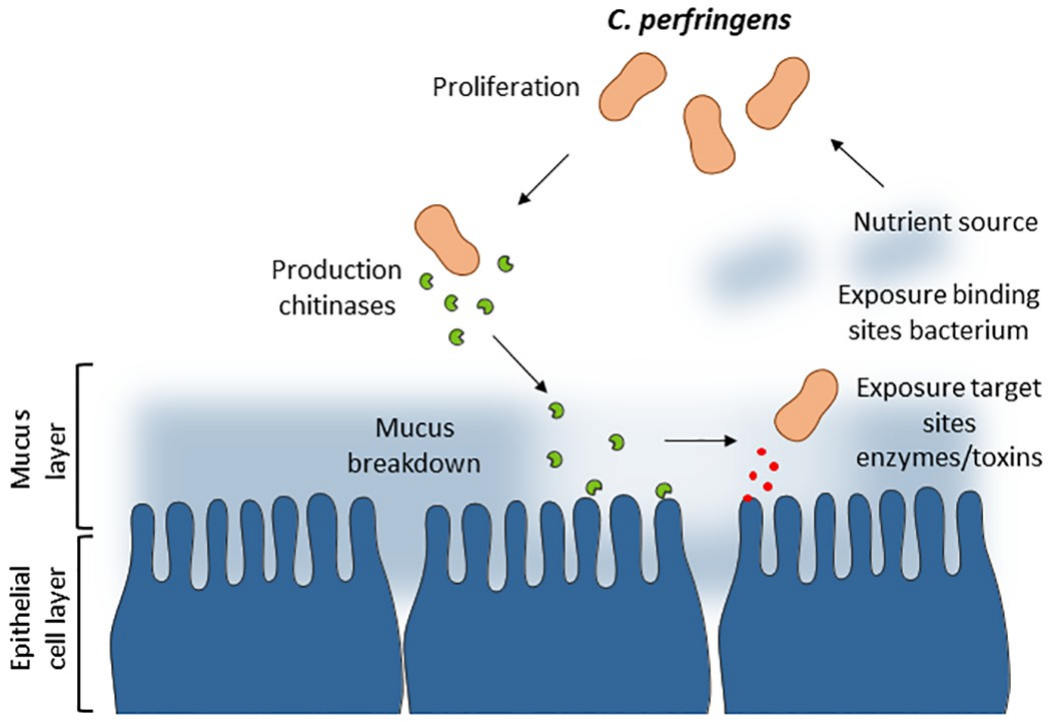
Graphic summary of the activity of pathogen-specific chitinases during pathogenesis. Virulent *C*. *perfringens* type G strains are in close proximity to the intestinal mucus layer. The production of chitinases results in the breakdown of the intestinal mucus layer that could result in nutrient acquisition, the exposure of additional binding sites for the bacteria and the exposure of target sites for toxins or other virulence factors (or a simultaneous combination). This aids additional *C*. *perfringens* proliferation and bacterial colonization, key aspects of early pathogenesis.

Mucus breakdown can result in nutrient acquisition, the exposure of additional binding sites for the bacteria and the exposure of target sites for toxins or other virulence factors (Fig 7). The potential role of chitinases in bacterial proliferation after mucus breakdown was simulated *in vitro* using multiple complementary growth assays using chicken intestinal mucus as the nutrient source. In the growth assay, chitinase mutant strains grow less in medium supplemented with mucus as compared to the wild-type strain. This effect could not be validated using the *in vitro* competition assay. Equal results were shown when wild type *C*. *perfringens* grew better in mucus that was pretreated with recombinant chitinase protein. The combined results indicate that the absence of chitinases (mainly ChiA) in the culture medium, either supplemented or produced by the bacteria itself, diminished *C*. *perfringens* growth significantly. In addition to nutrient acquisition, the importance of chitinases in bacterial attachment to the mucus layer was shown. Indeed, the binding of *C*. *perfringens* to mucus pre-treated with chitinase was higher as compared to untreated mucus. Furthermore, an *in vivo* colonization assay has shown that the chitinase mutant strain CP56Δ*chiA1* was less able to colonize the gastrointestinal tract as compared to the wild-type strain. These results indicate that chitinases are indeed important during the colonization and acquisition of nutrients by *C*. *perfringens* during NE pathogenesis. To further strengthen this statement, the biological significance of the chitinases during the overall pathogenesis was suggested during an *in vivo* NE trial. Indeed, disease caused by the chitinase mutant strain CP56Δ*chiA1* was less severe as compared to the wild-type strain. Additional research is required to further uncover the combined and individual role of both chitinases during mucus breakdown and eventually pathogenicity. Potentially, both chitinases are accessory virulence factors with ChiA breaking down larger branched structures whereas ChiB subsequently aids cleavage into metabolizable structures, or alternatively only one of two chitinases plays a significant role whereas the other is of minor importance during the pathogenesis, which is in accordance to the chitinases expressed by *L*. *monocytogenes* [41].

Inhibition of early colonization, through targeting of the chitinases is a promising new field to explore. Bisdionin C is known to target bacterial GH18 enzymes but could result in unspecific targeting [49]. A more specific approach using vaccination strategies could be more effective. Since a high mucosal antibody response (and not systemic response) is needed to protect animals against this disease, oral vaccination (for instance using non-pathogenic vaccine carriers) could be of great interest [50]. In addition, oral supplementation of nanobodies directed towards the chitinases could overcome the hurdle of active immunization [51].

In conclusion, our study has demonstrated that the pathogen-specific chitinases produced by *C*. *perfringens* type G strains are functional proteins and that they play accessory roles during pathogenesis through their ability to interact with the intestinal mucus layer. The subsequent mucus breakdown facilitates early bacterial colonization through enhanced bacterial proliferation and binding. Future research focussing on the inhibition of these enzymes could be of great interest in developing strategies to control this disease.

## Materials and methods

### Ethics statement

All experimental protocols were approved by the ethical committee of the Faculty of Veterinary Medicine, Ghent University (EC2019-5). The animal experiments were conducted in accordance with the approved protocols.

### Bacterial strains and culture conditions

*C*. *perfringens*: The previously described *C*. *perfringens* strain CP56 was used as the wild-type strain (Belgian Coordinated Collections of Microorganisms LMG 33101 [52,53]). Isogenic *C*. *perfringens* mutant strains were generated and validated as described below. In addition, a collection of *C*. *perfringens* isolate strains, obtained from different hosts, was studied (S5 Table). All *C*. *perfringens* strains were grown in an anaerobic chamber with atmosphere containing 10% H_2_, 10% CO_2_ and 80% N_2_ (Jacomex, Dagneux, France) at 42°C. Nutrient broths that were used are nutrient rich medium (Brain Heart infusion broth; BHI; Thermo Fisher, Merelbeke, Belgium), nutrient poor medium (50% tryptic soy broth, 25% nutrient broth and 25% peptone water [54]) and nutrient poor medium supplemented with 5% mucus.

*E*.*coli*: Both One Shot TOP10 *E*. *coli* (Invitrogen) and *E*.*coli* T7 Express lysY/Iq (New England Biolabs, Ipswich, USA) were used during the production of the recombinant chitinase proteins. *E*. *coli* CA434 was used as a donor strain during the production of the isogenic *C*. *perfringens* mutant strains. *E*.*coli* was either cultured in Terrific broth (12 g/l tryptone, 24 g/l yeast extract, 0.4% glycerol, 0.019 M KH_2_PO_4_ and 0.08 M K_2_HPO_4_) or Luria-Bertani Broth (Thermo Fisher) and incubated at 37°C.

### Generation of *C*. *perfringens* mutant strains

#### ClosTron mutagenesis system

Chitinase isogenic mutant strains were produced using the ClosTron mutagenesis system, as previously described [30]. In short, ClosTron intron-targeting regions were designed to insert at the 389 bp and 613 bp gene position for *chiA* (GenBank accession number F8UNI5) and *chiB* (GenBank accession number F8UNI4), respectively (S1 Data). The intron containing the target regions and inactivated antibiotic resistance gene were synthesized and cloned into ClosTron plasmid pMTL007C (ATUM, Newark, CA, USA). The chemically competent *E*. *coli* CA434 strain was transformed with the plasmid, serving as a conjugal donor strain [55]. The mixture, containing both the *E*.*coli* donor strain and *C*. *perfringens* recipient strain, was plated onto a non-selective Brain heart infusion broth (BHI) agar plate (Fisher Scientific, Merelbeke, Belgium) allowing conjugal transfer of the plasmid. Selective plating onto a BHI agar plate containing 30 U/ml polymyxin B sulphate (PMB), 100 μg/ml sodium sulfadiazine (SDZ) and 15 μg/ml thiamphenicol, favours growth of recipient strains containing the plasmid. Integration of the intron into the target site while losing the plasmid was selected through subsequent plating favouring erythromycin resistance and thiamphenicol sensitivity, respectively.

#### Evaluation of mutant strains

Mutant strains were validated for the presence of the on-target insertion of the ClosTron insert (through PCR) and the absence of additional off-target insertions (using dPCR), as previously described [30]. In short, the amount the ClosTron insert was quantified and compared to the amount of a reference gene, located in close proximity to the gene. An equal amount of both genes indicate the absence of off-target insertions and therefor validation of the mutant strain.

Several attempts were made to complement the mutant strains using the pJIR750 shuttle vector carrying either chitinase gene. However, these efforts were unsuccessful. Alternatively, each mutant (two for each chitinase) was evaluated extensively to assess whether their phenotype was the result of the targeted mutations or was hampered by additional secondary mutations.

First, whole genome sequencing of both wild-type and mutant strains was performed to assess potential secondary mutations of the mutant strains. *C*. *perfringens* DNA was extracted as previously described by Pitcher et al. [56], with some minor modifications as described by Dierick et al. [30]. Sequencing libraries were prepared using the TruSeq DNA PCR-free kit (Illumina) with an average insert size of 550 bp and sequenced using Illumina MiSeq v3 chemistry (2x 300 bp pair-end), both at Macrogen (Gasan-dong, World Meridian I, Seoul, South Korea). Quality filtering of the sequencing data was performed using Trimmomatic (v0.38, [57]) by cutting reads with an average quality per base below 15 using a 4-base sliding window and discarding reads with a minimum length of 200 bp, after which the quality was checked using the FastQC quality-control tool (Babraham Bioinformatics, Cambridge, UK). The CP56 quality-filtered reads were *de novo* assembled using SPAdes (v3.14.1) in isolate mode and short contigs (< 500 bp) were removed from the dataset [58]. The filtered CP56 assembly was annotated using PROKKA for use as a reference genome in variant analysis [59]. Single-nucleotide polymorphisms (SNPs) and insertion/deletions (INDELs) between CP56 and mutant strains were identified using the Genome Analysis Toolkit 4 (GATK4, v4.3.0), following the best practices for variant discovery analysis outlined by the Broad Institute [60]. More specifically, reads from the mutant strains were aligned against the CP56 reference genome using BWA-mem (v0.7.17-r1188, [61]) and duplicate reads identified with GATK *MarkDuplicatesSpark*. Variants were called per mutant strain using GATK *HaplotypeCaller* in GVCF mode. The resulting GVCFs files were imported into a GenomicsDB datastore, followed by joint genotyping on the GenomicsDB workspace using GATK *GenotypeGVCFs*. SNPs and INDELs were extracted using GATK *SelectVariants*, followed by quality filtering with GATK *VariantFiltration* according to GATK best practices: quality by depth “QD < 2.0”, Fisher strand “FS > 60.0”, strand odds ratio “SQR > 4.0”, read position rank sum test “ReadPosRankSum < -8.0”, mapping quality “MQ < 40.0” and mapping quality rank sum test “MQRankSum < -12.5”. For the remaining SNPs in each dataset, SNPEff (v5.1d) was used to annotate and predict variant effects [62]. Variants labeled as “High” or “Moderate” were further visualized and inspected in Integrated Genome Browser (v2.4.9, [63]).

Secondly, the growth properties of the mutant strains were evaluated to assess the impact of potential secondary mutations, as described under ‘Effect of mucus on growth properties of chitinase mutants’.

Lastly, the effect of secondary mutations on the NetB production of the *C*. *perfringens* strains was evaluated through an haemolytic assay, as described previously [64]. In short, 20% culture supernatant was incubated with 2% (v/v) chicken erythrocytes (diluted in HBSS-) in a 96-well plate. After an incubation period of 30 minutes at 37°C, intact red blood cells were pelleted and the released hemoglobin in the supernatant was quantified at OD_550nm_. A matched non-parametric Friedman test with Dunn’ multiple comparison (confidence interval 95%) was performed using GraphPad Prism 8 software.

### Preparation of colloidal chitin

Colloidal chitin was produced as previously described [65]. In short, 10 g chitin was diluted in 200 ml HCl. After overnight stirring at 4°C, 4 L water was added to the acidic solution. The colloidal chitin was collected after numerous repetitions of centrifugation (10,000 g, 15 min) and washing steps until a pH of 7 was reached.

### Mucus isolation from chicken small intestine

Chicken mucus was isolated from 20-day old coccidiosis-challenged broilers, since coccidiosis is a known predisposing factor to NE in broilers and could potentially affect mucus composition. Crude mucus was scraped off the inner lining of the small intestine (jejunum) and diluted 1/3 in 25 mM HEPES buffer (Sigma-Aldrich, pH 7.4). After five days of incubation at 4°C on a rotating shaker (100 rpm), the mixture was centrifuged and the obtained supernatant recentrifuged multiple times (twice 20 min at 4,700 g; 20 min at 20,000 g; twice 45 min at 20,000 g). The supernatant was filtered using 0.45 μm filters and stored at -20°C. Protein concentration levels were determined using the Pierce BCA Protein Assay Kit (Thermo Fisher). The isolated mucus samples were used individually as biological replicates in the dotblot and turbidity assay. In all other assays, every four mucus samples were pooled into larger samples.

### In Silico characterization of *C*. *perfringens* chitinases

#### Cellular localization and physiochemical properties

All analyses were performed using the translated amino acid sequences of the chitinase genes (GenBank accession number ChiA: F8UNI5 and ChiB: F8UNI4). Subcellular location was predicted using the web-based system CELLO2GO, with parameters specific for Gram-positive bacteria [66]. Signal peptide prediction was performed using the software tool SignalP version 5 (https://services.healthtech.dtu.dk/services/SignalP-5.0/ [67]), whereas prediction of non-classical protein secretion was performed using the software solution SecretomeP 2.0 (https://services.healthtech.dtu.dk/services/SecretomeP-2.0/ [68]). All tools were provided by the Centre for Biological Sequence Analysis, BioCentrum-DTU, Technical University of Denmark. In all subsequent analyses, the mature ChiA protein obtained after SignalP analysis, lacking its signal peptide, was used. The theoretical isoelectric point (pI) and molecular weight (MW), as well as various measures for protein stability were computed using the ExPASy ProtParam tool (https://web.expasy.org/protparam/). Overall sequence similarity between ChiB and the mature ChiA was calculated using protein-protein BLAST (NCBI). Proteins with similar domains (GH18 or GH19) were searched in published genome sequences of *C*. *perfringens* in the CAZy database (www.cazy.org, 13/08/2024). Additionally, the NCBI whole genome sequencing assembly database containing 778 complete genomes of *C*. *perfringens* (minimum scaffold or contig level; downloaded on 13/08/2024) was searched for GH18 (PFAM accession PF00704) or GH19 (PFAM accession PF00182) domain containing proteins using HMMER v3.3.2 (E-value cut-off = 1e-05). Overall sequence similarity between the identified GH18 domain containing proteins and ChiA or ChiB was calculated using protein-protein BLAST (NCBI).

### Prevalence of chitinase genes in *C*. *perfringens* collection

Using PCR, the prevalence of the chitinase genes *chiA* and *chiB* was assessed in *C*. *perfringens* strains obtained from an array of different hosts. In addition to many *netB*-negative type A and *netB*-positive type G strains from broilers, the collection also contained isolates obtained from layers, cows (suffering from enterotoxaemia or healthy calves and cattle), pigs, horses, goats, dogs, deer, sheep and humans. A complete list from these strains is provided in S5 Table. The fragments were PCR amplified using the BioMix DNA polymerase according to the manufacturers’ instructions (Bioline, London, UK). The primers that were used to amplify the *chiA* or *chiB* gene are given in Table 2 (ChiA_fw + ChiA_rev and ChiB_fw + ChiB_rev, respectively. The PCR reaction procedure consisted of: initial denaturation 3 min at 95°C, 35 amplification cycles (30 s at 95°C, 30 s at 50°C and 90 s at 72°C) and final elongation 12 min at 72°C.

**Table 2:** Primer sets. Primer sets used to amplify and clone chitinase genes (*chiA* or *chiB*) into the pBAD TOPO TA expression vector. Additional nucleotides (underlined) were added to incorporate an additional stop-codon (TGA), ribosome binding site (AGGA) and start-codon (ATG). Mutagenic primers used to preform site-directed mutagenesis at the active site of the chitinase genes *chiA* or *chiB*. Flanking primers are equal to the primers used to produce the enzymatically active enzymes. Nucleotide substitution of the mutant primers is indicated in the box.

Primer name	Sequence (5’ = > 3’)
ChiA_fw	GTTTCTTGAGAGGAATAATAAATGACAAAAGCTAAAGAAAAATTTAAAACA
ChiA_rev	GTTTCTTGGTTTATCATTACCAATTGGATTCATTCCATT
ChiB_fw	GTTTCTTGAGAGGAATAATAAATGAATACAATCTCTGTTAAGGCTATGAGT
ChiB_rev	GTTTCTTGGTGAATTTGTATTTTCCCAAATTGTTTGTCTATT
Enzymatically inactive enzymes:	
ChiA_rev_B	GGATGAAGGATATTGGAAAATCAATGTCTACTCC
ChiA_fw_C	GGAGTAGACATTGATTTTCAATATCCTTCATCC
ChiB_rev_B	GGTTCTCTGTAATCTCCAGGATAGTCCCAATCAATATC
ChiB_fw_C	GATATTGATTGGGACTATCCTGGAGATTACAGAGAACC

### Expression of chitinase genes using qPCR

An overnight bacterial culture of CP56 was diluted 1/1000 in fresh culture medium, either nutrient rich medium (BHI) or nutrient poor medium supplemented with 5% chicken intestinal mucus. RNA was extracted during the exponential phase using the Aurum Total RNA mini kit (Bio-Rad), according to manufacturer’s instructions. DNA was removed using the Turbo DNA-free kit (Invitrogen). cDNA was synthesized using the iScript cDNA synthesis kit (Bio-Rad). The expression of the chitinase genes was assessed using a SYBRgreen qPCR assay. Each 12 μl qPCR reaction consisted of 2μl template cDNA (30 ng), 6 μl SensiMix SYBR1 & Fluorescein Kit (Bioline), 0.5 μM forward primer and 0.5 μM reverse primer (*chiA*: FW 5’-gggtgggaaaatgttcaaggtgg-3’, REV 5’-gcaccagccggttctaaaactc-3’; *chiB*: FW 5’-gatgcagacctttctccaacgc-3’, REV 5’-ccatacaccaccagctcttcct-3’; *rpoA*: FW 5’-acatcattagcgttgtcagttaaag-3’, REV 5’-gaggttatggaataactcttggtaatg-3’). Cycling was performed on a real-time PCR thermal cycler (Biorad) and conditions were as follows: 95°C for 10 min, followed by 40 cycles of 95°C for 45 s and 60°C for 1 min. The fluorescent products were detected at the last step of each cycle. Data was analysed using the qBase+ software.

### Recombinant protein production

#### Recombinant production of native *C*. *perfringens* chitinases

The genes encoding either chitinase ChiA (mature protein by omitting the first 34 AA; signal peptide predicted by SignalP) or chitinase ChiB (full length) were cloned into the pBAD TOPO TA expression vector to construct proteins with a C-terminal HIS-tag (Invitrogen, Cergy Pontoise, France). The DNA sequences of both genes were PCR amplified using the velocity DNA polymerase (Bioline) using DNA obtained from the *netB*+ *C*. *perfringens* wild-type strain CP56 (Table 2). The forward primer contained an in-frame stop codon and translation re-initiation sequence to remove the plasmid-encoded N-terminal leader and allow native protein expression. The reverse primer excluded the native gene stop codon, resulting in the addition of the plasmid-encoded C-terminal V5 epitope and poly-histidine region for affinity purification (Table 2). The PCR reaction procedure consisted of: initial denaturation 2 min at 98°C, 35 amplification cycles (30 s at 98°C, 30 s at 54°C and 1 min at 72°C) and final elongation 10 min at 72°C. The resulting PCR product was cloned into the pBAD-TOPO expression vector, and transformed into One Shot TOP10 *E*. *coli* (Invitrogen), both according to manufacturer’s instructions, after which the correct orientation of the insert was verified by Sanger sequencing.

*E*.*coli* strains harbouring the pBAD-vector for chitinase production were cultured in Terrific Broth (TB) containing 100 μg/ml ampicillin at 37°C under rigorous shaking (125 rmp). At the mid-exponential phase, expression was induced by supplementation of the medium with 0.02% L-(+)-arabinose. Following overnight incubation, the cells were pelleted using centrifugation (10,000 g, 10 min, 4°C). The recombinant proteins were extracted from the pellet using the BugBuster protein extraction reagent (Merck, Overijse, Belgium) and purified on a Ni-Sepharose column (His GraviTrap, Cytiva, Hoegaarden, Belgium) both according to manufacturer’s instructions. Proteins were eluted using PBS containing 500 mM imidazole and 10% glycerol. The eluted solution was dialyzed against PBS containing 10% glycerol to get rid of the residual imidazole fraction.

The purity of the proteins was evaluated using SDS-page and Coomassie Brilliant Blue R-250 staining of the gel (S6 Fig). Protein concentration was quantified using the Pierce BCA Protein Assay Kit (Fisher Scientific, Brussel, Belgium). Purified proteins were stored at -20°C.

#### Recombinant production of enzymatically inactive chitinases

Enzymatically inactive enzymes were constructed using site-directed mutagenesis by a PCR-mediated method of extending overlapping gene segments with the desired mutation as previously described [69]. Therefore, the proton donor in the active site (Glutamate) was substituted with either aspartic acid (ChiB: E160D) or glutamine (ChiA: E196Q) using internal primers harbouring the AA substitution (Table 2). Although glutamine has the same structure as glutamate, it is not a proton donor. Changing glutamate to glutamine will not affect the structure of the active site cleft, but no enzymatic activity should be observed. Aspartate can act as a proton donor, but it is smaller than glutamate. Therefore, the proton is not in proximity to the substrate and no enzymatic cleavage of the substrate should occur. As described for the active enzymes, the resulting PCR product was cloned into the pBAD-TOPO expression vector, and transformed into One Shot TOP10 *E*. *coli*, after which the correct orientation of the insert was verified by Sanger sequencing. The resulting recombinant enzymes were produced as described above. Loss of enzymatic activity of the inactive recombinant chitinase enzymes was confirmed using the Chitinase Assay Kit as described under ‘Enzymatic activity towards pseudo-chitin substrates’. Either no activity was observed or k_cat_/K_m_ was reduced to less than 1% of its original value.

#### Recombinant production of truncated chitinase A for X-ray crystallography

In addition to the mature recombinant ChiA protein, a N-terminally trimmed version of ChiA (starting at residue S45 = domain boundary of the TIM domain) was designed to study the crystal structure of this protein more extensively. The trimmed gene construct followed by a poly-histidine tag was subcloned into the multiple cloning site (MCS) of a pET15b vector, by traditional restriction ligation. The pET15b plasmid was transformed into *E*.*coli* T7 Express lysY/Iq (New England Biolabs, Ipswich, USA) by heat-shock and selected on Luria-Bertani (LB)-agar plates supplemented with 100 μg/mL carbenicillin. Expression was induced with 0.8 mM IPTG after which cells were left to express overnight. Cells were harvested by centrifugation and lysed by sonication in HEPES-buffered saline (HBS, 25 mM HEPES, 150 mM NaCl pH 7.4). Supernatant containing ChiA was clarified by centrifugation followed by filtration. ChiA was captured from the supernatant by immobilized metal affinity chromatography (IMAC) followed by size exclusion chromatography (SEC) on a Superdex-200 column (Cytiva).

### Structural determination by X-ray crystallography

#### Additional protein purification

Protein crystal structures were studied using the N-terminally trimmed version of ChiA (starting at residue S45) and the full length ChiB recombinant protein. To study protein-ligand interactions, the active site point mutant E196Q of ChiA was used. Significant protein yields allowed us to polish the ChiA protein by batch recrystallization. To a few ml of ChiA at approximately 11 mg/ml an equal volume of precipitant mix (28% Broad PEG smear, 100 mM NaCl and 100 mM sodium phosphate pH 5.8) was gently added and mixed in. After equilibration, this mixture was seeded with a few single crystals harvested from a crystallization experiment leading to the formation of a crystal slurry in about one hour at room temperature. The crystals were collected by centrifugation at 700 g and dissolved in HBS. Three rounds of recrystallization yielded a final recovery of about 50% of highly pure ChiA. For the E196Q mutant of ChiA the protein was purified by IMAC followed by SEC only. For ChiB, purified protein as described above, was purified once more by a final SEC on a Superdex 200 increase column (Cytiva, Bornem, Belgium).

#### Crystallization and X-ray diffraction data collection and processing

All crystallization experiments were set up using a Mosquito crystallization robot (SPT Labtech, Melbourn, UK) with sitting drop vapor diffusion geometry in Swissci triple-drop plates. Screening for crystallization conditions was performed with commercial sparse matrix screen purchased from Hampton Research (Alisa Vieja, CA, USA), Molecular Dimensions (Rotherham, U.K.) and Rigaku (Tokyo, Japan). All crystals were vitrified in liquid nitrogen and datasets were collected at 100 K. Each of the X-ray diffraction datasets reported herein were collected from a single crystal and were scaled in XDS [70]. Detailed dataset statistics are presented in S2 Table. ChiA concentrated to 12 mg/ml readily crystallized in the presence of PEG with qualitative single crystals appearing in many conditions of the PEGION screen. All crystallization trials were set up via crystallization droplets consisting of 100 nl protein and 100 nl mother liquor (ML).

The ChiA Apo crystal grew in 0.2 M NaBr, 20% (w/v) PEG 3350 and was cryoprotected in ML with 15% (v/v) ethylene glycol prior to vitrification. Diffraction data were collected at the P14 beamline (PETRA III, EMBL C/O DESY, Hamburg, Germany) and processed in space group P2_1_2_1_2_1_ (a = 50.47 Å, b = 109.03 Å, c = 111.58 Å, α = β = γ = 90°) to a resolution of 1.50 Å.

The crystal of ChiA E196Q mutant in complex with its substrate chitin grew in 0.2 M NaF, 20% (w/v) PEG 3350, 1 mM chitin hexamer (Elicityl, Crolles, France). To limit substrate degradation crystallization was sped up by microseeding and crystals were harvested after a few hours. Crystals were cryoprotected in ML with 17% ZW2:2:1 (40% v/v DMSO, 40% v/v Ethylene Glycol, 20% v/v Glycerol) prior to vitrification [71]. Diffraction data were collected at the P14 beamline and processed in space group P2_1_2_1_2_1_ (a = 50.49 Å, b = 111.60 Å, c = 108.92 Å, α = β = γ = 90°) to a resolution of 1.35 Å.

The crystal of ChiA in complex with the chitinase inhibitor Bisdionin C grew in 0.2 M lithium acetate, 20% (w/v) PEG 3350 and was soaked in ML with 1 mM Bisdionin C (Sigma-Aldrich, Saint Louis, MO, USA). The crystal was cryoprotected in ML with 17% ZW2:2:1 prior to vitrification. Diffraction data were collected at the P14 beamline and processed in space group P2_1_2_1_2_1_ (a = 50.33 Å, b = 108.80 Å, c = 111.08 Å, α = β = γ = 90°) to a resolution of 1.35 Å.

The crystal of ChiA in complex with the chitinase inhibitor chitosan grew in 0.2 M LiCl, 20% (w/v) PEG 3350, 1 mM chitosan hexamer (Elicityl). The crystal was cryoprotected in ML with 17% ZW2:2:1 prior to vitrification. Diffraction data were collected at the P14 beamline and processed in space group P2_1_2_1_2_1_ (a = 50.44 Å, b = 108.50 Å, c = 111.41 Å, α = β = γ = 90°) to a resolution of 1.30 Å.

Screening for crystallization conditions for ChiB at 13 mg/ml did not yield obvious hits as was the case for ChiA. Sparse-matrix screens were also set up in the presence of 1 mM Anderson-Evans polyoxotungstate (TEW)(Jena Bioscience, Jena, Germany). In the presence of phosphate or lithium salts, flat hexagonal TEW crystals regularly appeared. One ChiB crystal did grow in condition E7 of the BCS screen (0.1 M Mg Formate 0.1 M RbCl 0.1 M PIPES pH 7 25% PEG Smear High), nucleating from precipitate. The crystal was cryoprotected in ML with 15% PEG400 prior to vitrification. Diffraction data were collected at the Proxima 2a beamline (Soleil, Saint-Aubin, France) and processed in space group C2 (a = 192.88 Å, b = 59.80 Å, c = 68.71 Å, α = γ = 90°, β = 108.18°) to a resolution of 1.60 Å. This crystal form could not be reproduced. TEW is not present in the final structure.

A more reproducible hit for ChiB was found to be Index screen condition F4, with larger crystals growing when an altered geometry of 100 nl protein + 200 nl ML was used. The crystal of ChiB in complex with the chitinase inhibitor Bisdionin C grew in 5 mM each of CoCl_2_ NiCl_2_ CdCl_2_ and MgCl_2_, 100 mM HEPES pH 7.5, 12% PEG 3350 and 1 mM Bisdionin C. The crystal was cryoprotected in ML with 20% ethylene glycol prior to vitrification. Diffraction data were collected at the P14 beamline (EMBL Hamburg, PETRA III, Hamburg, Germany) and processed in space group P4_1_2_1_2 (a = b = 67.40 Å, c = 355.82 Å, α = β = γ = 90°) to 1.85 Å resolution.

#### Model building and crystallographic refinement

All reported crystal structures herein were phased by molecular replacement (MR) [72]. Iterative model building and refinement were performed in Coot [73], Buster [74] and Phenix.refine [75]. Detailed refinement statistics can be found in S2 Table. To phase a preliminary ChiA dataset, we identified the TIM barrel of the *Chromobacterium violaceum* chitinase by sequence identity (42% identity) as most similar structure present in de Protein Data Bank (PDB 4txg.) An interpretable MR solution was found with this search model. The carbohydrate binding domain was built de-novo. Further model building and refinement were performed as described above. All ChiA structures reported herein were phased with this experimentally determined ChiA model. Atomic displacement parameters for the ChiA structures were refined allowing for anisotropic B-factors for the individual non-hydrogen atoms.

Geometric restraints for the Bisdionin C molecule were generated in Grade [76]. To phase the ChiB dataset in spacegroup C2, we utilized the TIM barrel of ChiA as no more identical (29% identity between the ChiA and ChiB TIM domains) structures were available in the PDB. The phasing solution was further autobuilt with Phenix.autobuild [77]. Further model building and refinement were performed as described above. The obtained model of unbound Apo ChiB was used to phase the dataset of ChiB in complex with Bisdionin C. Atomic displacement parameters for the ChiB structures were refined allowing for group based anisotropic movement described by the TLS-model. Bisdionin C bound ChiB was submitted as a protein-ligand prediction target for CASP15 (target T1188).

### Substrate binding

To avoid enzymatic breakdown of the substrate, the enzymatically inactive recombinant enzymes were used for all binding assays.

#### In solution binding assay

Binding of the inactive recombinant enzymes to various substrates (chitin (Sigma-Aldrich), colloidal chitin and GlcNAc coated beads (Sigma-Aldrich)) was assessed using an in solution binding assay. The recombinant proteins (60 μg/ml) were added to 30 mg/ml substrate (chitin, colloidal chitin and GlcNAc coated beads), diluted in chitin binding buffer (50 mM Tris-HCl (pH 8), 1 mM EDTA (pH 8), 500 mM NaCl and 0.1% Tween80) and incubated for three hours at 4°C. The unbound (supernatant) and bound (pellet) protein fractions were collected after centrifugation and visualized using SDS-PAGE and subsequent Coomassie Brilliant Blue R-250 staining. Gels were scanned with a GS-800 calibrated densitometer (Bio-Rad, Temse, Belgium), after which the optical density per mm^2^ of the obtained bands was quantified using Quantity One software (Bio-Rad). Chitinase binding was expressed as the ratio of the bound protein to the total protein (intensity of the bound + unbound protein bands). The assay was performed in duplicate to provide technical replicates.

#### Dotblot

The binding properties of the inactive recombinant enzymes to porcine mucins (Type II and III, Sigma-Aldrich) and crude chicken mucus (isolated from small intestine) was assessed using a dotblot assay. The dotblot assay was performed by spotting and air drying 20 μg of mucus onto a nitrocellulose membrane. Positive controls (25 μg of recombinant enzyme) and negative controls (PBS) were included in the assay. Isolated chicken mucus from six different birds was included to provide biological replicates and performed in duplicate. Non-specific binding was blocked through the incubation of the membrane with 5% BSA in PBS-T buffer (PBS containing 0.05% Tween-20, pH 7.4) for one hour at room temperature. Afterwards, the membrane was incubated with 100 μg/ml of either recombinant enzyme or PBS (as a negative control) diluted in TBS-T buffer containing 0.1% BSA. Membranes were washed three times for 5 min with PBS and incubated with the secondary anti-HIS antibody conjugated with HRP (Sigma-Aldrich) for one hour at room temperature. After extensive washing, the amount of bound protein was visualized using the Pierce DAB visualization kit (Thermo Fisher), according to manufacturer’s instructions.

### Enzymatic activity

#### Enzymatic activity towards chitin

The enzymatic activity of the chitinases towards chitin was assessed using the 3-methyl-2-benzothiazolinone hydrazone (MBTH) assay through the quantification of the amount of reducing sugar ends. The MBTH assay was performed as previously described [78]. In short, 30 μg/ml of active recombinant enzyme was added to 0, 1, 2, 5, 7.5, 10, 20 or 30 mg/ml colloidal chitin (produced as described above) diluted in PBS (pH 5). After a one hour incubation period at 37°C, the reaction was stopped by adding an equal amount of 0.5 N NaOH. The MBTH mixture (0.5 mg/ml 1,4-Dithiothreitol, 1.5 mg/ml 3-Methyl-2-benzothiazolinone-hydrazonehydrochloride) was added to the sample in a 1:2 ratio. After heating for 15 min at 80°C, the iron mixture (0.5% ammonium iron(III) sulphate, 0.5% sulfamic acid, 0.25N HCl) was added at a 2:3 volume ratio. After cooling, the absorbance was measured at 620 nm. Using a standard curve of GlcNAc, the corresponding amount of reducing ends in the mixture was calculated. A negative control sample was included for each substrate concentration, which was subtracted from the corresponding sample values to eliminate background. The experiment was conducted in triplicate. Using the GraphPad Prism 8 software, a theoretic Michaelis Menten curve was fitted to the obtained data and kinetic parameters (maximal velocity V_max_ and Michaelis Menten constant K_m_) of the fitted models were obtained. The catalytic constant k_cat_ was calculated as the ratio of V_max_ on the total enzyme concentration. The molecular weight of colloidal chitin was considered 920,000 g/mol for further calculations [79].

#### Enzymatic activity towards pseudo-chitin substrates

The chitinolytic activity of the recombinant chitinases was assessed using a fluorometric Chitinase Activity Kit (Sigma-Aldrich) according to the manufacturer’s instructions. In short, the enzymatic hydrolysis of three fluorescently labelled pseudo-chitin substrates (4-methylumbelliferyl N,N’-diacetyl-β-D-chitobioside (4-MU-GlcNAc), 4-methylumbelliferyl N-acetyl-β-D-glucosaminide (4-MU-GlcNAc_2_) and 4-methylumbelliferyl N-acetyl-β-D-N-N’-N””-triacetylchitriose (4-MU-GlcNAc_3_)) was assessed, being a measure for the enzymatic β-N-acetylglucosaminidase, chitobiosidase and endochitinase activity. The active recombinant enzymes (2.5 mg/ml for all conditions and 25 mg/ml for ChiA using 4-MU-GlcNAc_3_ since no activity was measured using lower enzyme concentrations) were added to the substrate (0, 0.00625, 0.0125, 0.025, 0.05, 0.1, 0.2 or 0.4 mg/ml, diluted in phosphate-citrate buffer pH 5) and incubated for 30 min at 37°C (total reaction volume 100 μl). The reaction was stopped by adding 200 μl 0.1 M sodium carbonate to each well. The amount of liberated 4-methylumbelliferone (4-MU) was quantified fluorometrically at an excitation wavelength of 360 nm and emission wavelength of 450 nm. Through the inclusion of a 4-MU standard curve, the hydrolysis rate of the reactions was calculated. The model for substrate inhibition was fitted to the data and kinetic parameters (maximal velocity V_max_ and Michaelis-Menten constant K_m_) were obtained using the GraphPad Prism 8 software. The catalytic constant k_cat_ was calculated as the ratio of V_max_ on the total enzyme concentration. The molecular weights of the substrates were considered 582.55 and 785.75 g/mol for 4-MU-(GlcNAc)_2_ and 4-MU-(GlcNAc)_3_, respectively for further calculations.

#### Effect of pH and temperature on the enzymatic activity

The effect of temperature and pH was assessed using the above described chitinase activity assay towards the pseudo-chitin substrates 4-MU-GlcNAc_2_ and 4-MU-GlcNAc_3_. A temperature interval of 5–42°C was evaluated at a constant pH of 5, the pH interval [4–7] at a constant temperature of 42°C with the constant parameters representing the theoretical physiological conditions inside the chicken intestine. The optimal combination of substrate and enzyme concentration was chosen at which the activity towards the labelled pseudo-chitin substrates was highest: 0.025 mg/ml 4-MU-GlcNAc_2_ for 2.5 μg/ml ChiB; 0.01 mg/ml 4-MU-GlcNAc_3_ for 2.5 μg/ml ChiB; 0.4 mg/ml 4-MU-GlcNAc_2_ for 2.5 μg/ml ChiA and 0.4 mg/ml 4-MU-GlcNAc3 for 25 μg/ml ChiA.

#### Enzymatic activity towards chicken mucus

The effect of the recombinant chitinases towards chicken mucus was assessed using a turbidity assay. 15 μg of the active recombinant enzyme was added to 150 μg of crude chicken mucus (isolated as described above, 12 biological replicates) in a total volume of 150 μl phosphate-citrate assay buffer (pH5). After a one hour incubation period at 37°C, the turbidity of the mixture was measured at an OD-value of 450 nm. Crude chicken mucus without the addition of chitinase was used as a negative control. The relative index was calculated by dividing the OD of the chitinase-treated mucus by the OD of the untreated mucus sample for each mucus sample. A non-parametric paired Friedman test including Dunn’s multiple comparison with a confidence interval of 95% was applied using the GraphPad Prism 8 software.

### Biological significance of chitinases during *C*. *perfringens* proliferation

#### Effect of chitinase-treated intestinal mucus on *C*. *perfringens* growth

150 μg of chicken intestinal mucus was pre-treated with 15 μg of either recombinant ChiA or ChiB in a total volume of 150 μl phosphate-citrate assay buffer (pH 5). After an incubation period of one hour at 37°C, the mucus was diluted in 1/5 in a total volume of 1 ml HBSS+, after which a 1/1000 dilution of overnight culture of wild type CP56 was added to the mixture. For each sample, three wells of a 96-well plate were filled with 200 μl of the bacterial culture (technical triplicate) inside the anaerobic chamber and tightly sealed to inhibit air influx. The optical density at 600 nm was measured semi-continuously (every 5 min for a total of 6 hours) using the Multiscan Microplate photometer with integrated incubator (42°C, continuous shaking) (Thermo Fisher). The growth rate and saturation level were determined as the slope during the exponential phase and maximal OD value, respectively. The analysis was conducted in triplicate using different overnight cultures (biological triplicates). A matched non-parametric Friedman test with Dunn’ multiple comparison (confidence interval 95%) was performed using GraphPad Prism 8 software.

#### Effect of mucus on growth properties of chitinase mutants

The ability of wild type (CP56) and mutant *C*. *perfringens* strains (CP56Δ*chiA1*, CP56Δ*chiA2*, CP56Δ*chiB1* and CP56Δ*chiB2*) to use chicken mucus as a substrate, was assessed using growth analysis. In short, overnight cultures were 1/1000 diluted in different media: nutrient rich medium (BHI), nutrient poor medium and nutrient poor medium supplemented with 5% crude chicken mucus. The optical density at 600nm was measured semi-continuously, as described above. The ratio of 1 divided by the amount of time needed to reach the middle of the logarithmic phase was calculated and compared using a one-way ANOVA with Sidak’s multiple comparison (confidence interval 95%) using GraphPad Prism 8 software.

#### *In vitro* competition assay

Individual overnight cultures of wild type CP56 and mutant strain (CP56Δ*chiA1* or CP56Δ*chiB1*) were mixed in a 1:1 ratio. This mixture was used to inoculate fresh nutrient rich medium (BHI) or nutrient poor medium supplemented with 5% chicken mucus in a 1/1000 dilution and incubated in an anaerobic chamber. During different stages of growth (middle exponential phase OD600~0.8, saturation = overnight incubation), DNA was isolated from the mixed culture. For this, 200μl of the bacterial culture was centrifuged (12,000g, 5min) after which the pellet was resuspended in 40μl lysis buffer (0.2 M NaOH, 1% SDS). Next, the mixture was heated 5min at 95°C after which 9 volumes of water were added to dilute the sample. The amount of wild-type and mutant strain was determined using digital PCR as described previously [30]. The competition index was calculated as the ratio of mutant to wild-type strain divided by its value at inoculation. The experiment was performed in triplicate. Normality was checked using the Kolmogorov-Smirnov test. The average index at different stages of growth was compared to a theoretical value of 1 using a one-side t-test (confidence interval 95%) using the GraphPad Prism 8 software.

### Biological significance of chitinases during *C*. *perfringens* colonization

#### *In vitro* bacterial binding assay to intestinal mucus

The effect of the chitinase proteins on the adherence of wild type *C*. *perfringens* CP56 was assessed using a mucus binding assay [80,81]. Wells of a 24-well plate were coated with a thin layer (600 μl) of 1% agar containing chicken intestinal mucus in a 1:1 ratio. Control wells lacking mucus (substitution with PBS) were used to correct for potential non-specific binding. *C*. *perfringens* overnight culture was washed twice with PBS, after which 500 μl of washed culture was added to the wells, supplemented with either 50 μg of recombinant enzyme (ChiA or ChiB) or an equal volume of PBS as a negative control. Wells were anaerobically incubated for 90 min at 37°C on a rotary shaker (50 rpm). Afterwards, wells were washed five times with sterile PBS to wash away unbound bacteria. The agar layer was transferred to a falcon containing 5 ml of PBS using a sterile spoon. After 3min of vortexing, the mixture was plated onto BHI plates using a 10-fold serial dilution in PBS. Plates were incubated overnight in an anaerobic chamber at 42°C. The percentage of bound bacteria to the mucus was calculated using the following formula:

$$\%\;Bound\;bacteria\;to\;mucus=\frac{Bound\;bacteria\;to\;mucus-Bound\;bacteria\;in\;control}{Bacteria\;in\;inoculum}$$

The binding ratio is defined as the percentage of bound bacteria in the supplemented sample divided by this amount when untreated mucus was used. The assay was performed five times, using five different biological replicates of pooled intestinal mucus and different overnight cultures of the *C*. *perfringens* strain. Normality was checked using the Kolmogorov-Smirnov test. The average binding ratio was compared to a theoretical value of 1 (no effect of chitinase-treatment on *C*. *perfringens* binding to chicken mucus) using a one-side t-test (confidence interval 95%) using the GraphPad Prism 8 software.

#### *In vivo* colonization assay

As a result of the binding preference and enzymatic activity of ChiA toward mucus and not ChiB, only mutant CP56Δ*chiA1* was used in the following *in vivo* experiments. Ten male Ross 308 broiler chickens were housed in a pen of one square meter. Water and feed were supplied *ad libitum*. The feed was a wheat/rye-based (43%/7.5%) diet containing soybean meal as a protein source that was replaced by fishmeal (30%) from day 17 on. A tenfold dose of Paracox-5 (MSD Animal Health) or Evalon (MSD Animal Health, Boxmer, The Netherlands) was orally administered at day 14 and 16, respectively. On day 18, birds were inoculated with one millilitre of a mixed culture of CP56 wild-type and mutant strain in a 1:1 ratio (total amount of bacteria 10^9^ cfu/ml). One day later, birds were euthanized using an overdose of pentobarbital. Intestinal content of both jejunum and ileum was collected and stored at -20°C. The competition index was determined and analysed as described during the *in vitro* competition assay.

#### *In vivo* necrotic enteritis trial

An *in vivo* NE trial was conducted as previously described [82]. In short, 180 male Ross 308 broilers were housed in the same stable and divided into 12 equal groups (two conditions; six replicates per condition). Each group was housed with a density of 15 birds per square meter. Water and feed were supplied *ad libitum*. The feed was a wheat/rye-based (43%/7.5%) diet containing soybean meal as a protein source. Soybean meal was replaced by fishmeal (30%) from day 17 on, as a source of dietary animal protein, which is a known predisposing factor for induction of NE. A tenfold dose of Paracox-5 (MSD Animal Health) or Evalon (MSD Animal Health) was orally administered at day 14 and 16, respectively. At days 18 and 19, birds were challenged by oral administration of one millilitre overnight culture in BHI of either the wild-type *C*. *perfringens* strain CP56 or the mutant strain CP56Δ*chiA1* (total amount of bacteria 10^9^ cfu/ml). At day 20, birds were euthanized. At necropsy, the lesions in the duodenum, jejunum and ileum were scored using a well-established scoring system [83]. In short, score 0: no gross lesions; score 1: thin or friable walls, score 2: focal necrosis and ulceration (1–5 foci); score 3: focal necrosis and ulceration (6–15 foci); score 4: focal necrosis and ulceration (16 or more foci); score 5: patches of necrosis 2 to 3 cm long and score 6: diffuse necrosis. Due to its subjective nature, score 1 was not assigned. Disease severity scores were analysed with a cumulative link mixed model (clmm), using disease severity score (ordinal factor 0–6) as the response variable and *C*. *perfringens* strain (CP56 or CP56Δ*chiA1*) as a predictor variable, thereby accounting for non-independence of birds housed within the same pen by specifying “pen” as a random effect (*Ordinal* package [84] in R v4.2.1 [85]). Graphic representations of the data were designed using the *ggplot2* package [86].

## Supporting information

S1 Table Subcellular localization and secretion pathway prediction of ChiA or ChiB. (PDF)

S2 Table Physicochemical parameters of *C*. *perfringens* chitinases ChiA and ChiB computed using the ExPASy ProtParam tool. (PDF)

S3 Table Dataset and refinement statistics of X-ray crystallography. (PDF)

S4 Table GH18 domain containing proteins in *netB-*negative *C*. *perfringens* strains. (PDF)

S5 Table Presence of *chiA* and *chiB* in collection of *C*. *perfringens* strains using PCR screening. (PDF)

S6 Table Variants detected in CP56 chitinase mutants. (PDF)

S7 Table Variants detected in CP56 chitinase mutants and BLASTp results with predicted impacts. (PDF)

S1 Fig Overview of DNA sequence of chitinases ChiA and ChiB with annotated features and primers. (PDF)

S2 Fig Expression of chitinase genes using qPCR. (PDF)

S3 Fig Structure based alignments of *C*. *perfringens* ChiA and ChiB with related chitinases. (PDF)

S4 Fig NetB activity in culture supernatant of *C*. *perfringens* strains (wild-type and mutant strain) towards chickens erythrocytes. (PDF)

S5 Fig Growth analysis mutant strains in different culture media. (PDF)

S6 Fig Purity of recombinant ChiA and ChiB using Coomassie staining of SDS-page gel. (PDF)

S1 Data Nucleotide and protein sequence of chitinases ChiA and ChiB. (PDF)

S2 Data Source data for graphs in this study. (XLSX)
