# Supplementary material for: Clostridium perfringens chitinases, key enzymes during early stages of necrotic enteritis in broiler chickens
Source: PLoS Pathog. 2024 Sep 16;20(9):e1012560. doi: 10.1371/journal.ppat.1012560 (PMC11426533; doi:10.1371/journal.ppat.1012560)
Supplement: S1 Table — (PDF) [file ppat.1012560.s001.pdf]

S1 Table: Subcellular localization and secretion pathway prediction of ChiA or ChiB

	ChiA		ChiB	
**Cello predictions**	Score	Probability	Score	Probability
Extracellular	4.875	97.5%	3.840	76.8%
Cell wall	0.024	0.5%	0.224	4.5%
Membrane	0.029	0.6%	0.586	11.7%
Cytoplasmic	0.072	1.4%	0.350	7.0%
**SecretomeP predictions**	Score		Score	
SecP score	0.892		0.897	
**SignalP predictions**	Probability		Probability	
Signal peptide (Sec/SPI)	91.1%		10.4%	
TAT Signal peptide (Tat/SPI)	0.8%		0.2%	
Lipoprotein signal peptide (Sec/SPII)	5.0%		0.8%	
Other	3.2%		88.6%	
