# Supplementary material for: Clostridium perfringens chitinases, key enzymes during early stages of necrotic enteritis in broiler chickens
Source: PLoS Pathog. 2024 Sep 16;20(9):e1012560. doi: 10.1371/journal.ppat.1012560 (PMC11426533; doi:10.1371/journal.ppat.1012560)
Supplement: S2 Table — (PDF) [file ppat.1012560.s002.pdf]

S2 Table: Physicochemical parameters of *C. perfringens* chitinases ChiA and ChiB computed using the ExPASy ProtParam tool

	Number of amino acids	MW (kDa)	pI	Instability index	Aliphatic index	GRAVY
**ChiA**	577	65.35	5.16	28.98	74.14	-0.528
**ChiB**	599	65.91	4.84	27.86	72.79	-0.508
