# Supplementary material for: Clostridium perfringens chitinases, key enzymes during early stages of necrotic enteritis in broiler chickens
Source: PLoS Pathog. 2024 Sep 16;20(9):e1012560. doi: 10.1371/journal.ppat.1012560 (PMC11426533; doi:10.1371/journal.ppat.1012560)
Supplement: S3 Table — (PDF) [file ppat.1012560.s003.pdf]

S3 Table: Dataset and refinement statistics of X-ray crystallography

	ChiA				ChiB	
Name	Apo	Chitin hexamer	Bisdionin C	Chitosan hexamer	Apo	BisdioninC
Construct	S46-K611	S45-K611 E196Q	S46-K611	S46-K611	M1-S599	M1-S599
PDB code	8otb	8oye	8ose	8owf	8ovr	8c6z
**Dataset Statistics**						
Collection source	EMBL P14	EMBL P14	EMBL P14	EMBL P14	Soleil PX2	EMBL P14
Crystallization condition	0.2M NaBr, 20% (w/v) PEG 3350	0.2M NaF, 20 % (w/v) PEG 3350, 1mM (b-GlcNAc)6	0.2M lithium acetate, 20% (w/v) PEG 3350, 1mM BisdioninC	0.2 M LiCl, 20% (w/v) PEG 3350, 1mM (b-GlcN)6	0.1 M MgFormate 0.1 M RbCl 0.1 M PIPES pH 7 25% PEG Smear High	5mM $CoCl_2$ $NiCl_2$ $CdCl_2$ $MgCl_2$ (each),100mM HEPES pH7.5, 12% PEG 3350, 1mM BisdioninC
cryoprotection	15% EtGlyc	17% ZW2:2:1	17% ZW2:2:1	18% ZW2:2:1	15% PEG400	20% EtGlyc
Wavelength (Å)	1.033	0.976	1.033	0.976	0.980	0.976
Spacegroup	$P2_12_12_1$	$P2_12_12_1$	$P2_12_12_1$	$P2_12_12_1$	C2	$P4_12_12$
Unit cell (Å)	50.47 109.03 111.58	50.486 111.599 108.916	50.329 108.802 111.08	50.436 108.498 111.407	192.876 59.803 68.707	67.401 67.401 355.823
(°)	90 90 90	90 90 90	90 90 90	90 90 90	90 108.176 90	90 90 90
Resolution (Å)	37.43 - 1.50 (1.54 - 1.50)	77.95 - 1.35(1.43 - 1.35)	49.47 - 1.35 (1.39 - 1.35)	45.74 - 1.30 (1.38 - 1.30)	65.28 - 1.60 (1.69 - 1.60)	58.6 - 1.85 (1.96 - 1.85)
Unique refelction	97204 (5986)	134505 (20892)	133165 (9641)	148328 (23194)	98121 (15492)	70266 (11256)
Multiplicity	9.78 (7.69)	12.01 (6.96)	5.78 (5.45)	3.77 (3.31)	6.12 (6.05)	6.8 (6.81)
Completeness (%)	97.9 (82.4)	99.3 (96.3)	99.1 (98.1)	98.5 (96.5)	99.5 (98.0)	98.0 (99.1)
I/s	36.29 (3.3)	23.82 (3.40)	18.90 (2.38)	14.07 (1.31)	15.08 (1.73)	12.61 (1.2)
Rmeas (%)	3.4 (62.5)	5.8 (59.7)	4.4 (84.4)	5.5 (115.5)	7.2 (92.0)	10.0 (141.3)
CC(1/2) (%)	100.0 (82.2)	99.9 (86.1)	99.9(73.9)	99.9 (45.0)	99.9 (73.4)	99.9 (35.6)
Wilson B (Å²)	20.1	17.7	17.25	15.71	22.02	38.48
**Refinement Statistics**						
Resolution	34.43 - 1.50 (1.54 - 1.50)	77.95 - 1.35 (1.38 - 1.35)	49.47 - 1.35 (1.38 - 1.35)	45.74 - 1.30 (1.33 - 1.30)	45.09 - 1.60 (1.64 - 1.60)	48.94 - 1.85 (1.89 - 1.85)
R_{work} (%)	15.76 (19.16)	14.72 (26.33)	13.86 (19.83)	14.51 (26.56)	15.34 (28.65)	19.65 (34.89)
R_{free} (%)	17.54 (22.46)	16.12 (27.30)	16.30 (24.11)	16.98 (27.37)	17.16 (30.16)	22.34 (35.17)
Number of non-hydrogen atoms						
Protein	4618	4595	4599	4521	4493	4463
Ligand/ion	37	286	95	142	105	61
Water	576	576	633	687	661	416
B-factors (Å²)						
Protein	25.94	25.09	23.48	22.03	26.8	39.3
Ligand/ion	25.66	22.27	22.24	25.07	44.12	60.99
Water	36.75	36.62	33.34	35.83	36.3	42.74
Ramachandran favored (%)	96.79	96.08	97.33	96.79	97.72	98.07
allowed (%)	3.21	3.74	2.67	3.21	2.28	1.93
outliers (%)	0	0.18	0	0	0	0
R.m.s. deviations						
Bond lenght (Å)	0.004	0.004	0.01	0.006	0.011	0.012
Bond angles (°)	0.761	0.844	1.115	0.851	1.116	1.185
Molprobity score	1.22	1.18	1.04	1.18	0.94	0.96
