# Supplementary material for: Clostridium perfringens chitinases, key enzymes during early stages of necrotic enteritis in broiler chickens
Source: PLoS Pathog. 2024 Sep 16;20(9):e1012560. doi: 10.1371/journal.ppat.1012560 (PMC11426533; doi:10.1371/journal.ppat.1012560)
Supplement: S4 Table — (PDF) [file ppat.1012560.s004.pdf]

S4 Table: GH18 domain containing proteins in *netB*-negative *C. perfringens* strains.

The NCBI whole genome sequencing assembly database containing 778 complete genomes of *C. perfringens* (minimum scaffold or contig level; downloaded on 13/08/2024) was searched for GH18 (PFAM accession PF00704) or GH19 (PFAM accession PF00182) domain containing proteins using HMMER v3.3.2 (E-value cut-off = 1e-05). 85.7% (48/56) of the *C. perfringens* strains harbouring GH18, also contained NetB. The table represents the 8 strains that harbour a GH18 family member, but where no NetB was found. Overall sequence similarity between the identified GH18 domain containing proteins and ChiA or ChiB was calculated using protein-protein BLAST (NCBI).

Strain	GenBank accession No.	Host	Location	Protein ID	BLASTp ChiA		BLASTp ChiB	
					Query cover	Identity	Query cover	Identity
**G5**	GCF_032250115.1	Goat	China	WP_012748796.1	47.00%	20.60%	40.00%	26.59%
**x94**	GCF_034420755.1	manure treated soil from research farm	Canada	WP_322395987.1	19.00%	25.62%	24.00%	23.19%
**JIR246**	GCF_951336965.1	Porcine	Belgium	WP_163243987.1	no similarity		no similarity	
**CW500**	GCF_951337405.1	Porcine	unknown	WP_033860799.1	16.00%	25.66%	no similarity	
**CperBRD5**	GCF_951336235.1	Chicken	Australia	WP_283676337.1	43.00%	29.04%	43.00%	98.85%
**CperB2H112**	GCF_951336145.1	Chicken	Australia	WP_283697426.1	73.00%	29.03%	99.00%	95.83%
				WP_283697427.1	99.00%	96.07%	71.00%	29.75%
**Cper103980**	GCF_951336185.1	Chicken	Australia	WP_283697426.1	73.00%	29.03%	99.00%	95.83%
				WP_283697427.1	99.00%	96.07%	71.00%	29.75%
**CperB1H119**	GCF_951336835.1	Chicken	Australia	WP_283697426.1	73.00%	29.03%	99.00%	95.83%
				WP_283697427.1	99.00%	96.07%	71.00%	29.75%
