# Supplementary material for: Clostridium perfringens chitinases, key enzymes during early stages of necrotic enteritis in broiler chickens
Source: PLoS Pathog. 2024 Sep 16;20(9):e1012560. doi: 10.1371/journal.ppat.1012560 (PMC11426533; doi:10.1371/journal.ppat.1012560)
Supplement: S5 Table — (PDF) [file ppat.1012560.s005.pdf]

S5 Table: Presence of *chiA* and *chiB* in collection of *C. perfringens* strains using PCR screening.

A diverse collection of *C. perfringens* strains, obtained from different hosts, was screened using PCR to assess the prevalence of *chiA* and *chiB*. The fragments were PCR amplified using the BioMix DNA polymerase according to the manufacturers' instructions (Bioline, London, UK). The primers that were used to amplify the *chiA* or *chiB* gene are ChiA_fw (5'-GTTTCTTGAGAGGAATAATAAATGACAAAAGCTAAAGAAAAATTTAAAACA-3') + ChiA_rev (5'-GTTTCTTGGTTTATCATTACCAATTGGATTCATTCCATT-3') and ChiB_fw (5'- GTTTCTTGAGAGGAATAATAAATGAATACAATCTCTGTTAAGGCTATGAGT-3') + ChiB_rev (5'-GTTTCTTGGTGAATTTGTATTTTCCCAAATTGTTTGTCTATT-3'), respectively. The PCR reaction procedure consisted of: initial denaturation 3 min at 95°C, 35 amplification cycles (30 s at 95°C, 30 s at 50°C and 90 s at 72°C) and final elongation 12 min at 72°C.

Strain	Host	Disease state	*netB*	*chiA*	*chiB*	Location	Reference
CP1	Broiler	Healthy	-	-	-	Belgium	doi: 10.1016/j.vetmic.2005.10.023.
CP2	Broiler	Healthy	-	-	-	Belgium	doi: 10.1016/j.vetmic.2005.10.023.
CP3	Broiler	Healthy	-	-	-	Belgium	doi: 10.1016/j.vetmic.2005.10.023.
CP4	Broiler	Healthy	-	-	-	Belgium	doi: 10.1016/j.vetmic.2005.10.023.
CP5	Broiler	Healthy	-	-	-	Belgium	doi: 10.1016/j.vetmic.2005.10.023.
CP6	Broiler	Healthy	-	-	-	Belgium	doi: 10.1016/j.vetmic.2005.10.023.
CP7	Broiler	Healthy	-	-	-	Belgium	doi: 10.1016/j.vetmic.2005.10.023.
CP8	Broiler	Healthy	-	-	-	Belgium	doi: 10.1016/j.vetmic.2005.10.023.
CP9	Broiler	Healthy	-	-	-	Belgium	doi: 10.1016/j.vetmic.2005.10.023.
CP10	Broiler	Healthy	-	-	-	Belgium	doi: 10.1016/j.vetmic.2005.10.023.
CP11	Broiler	Healthy	-	-	-	Belgium	doi: 10.1016/j.vetmic.2005.10.023.
CP12	Broiler	Healthy	-	-	-	Belgium	doi: 10.1016/j.vetmic.2005.10.023.
CP13	Broiler	Healthy	-	-	-	Belgium	doi: 10.1016/j.vetmic.2005.10.023.
CP15	Broiler	Healthy	-	-	-	Belgium	doi: 10.1016/j.vetmic.2005.10.023.
CP17	Broiler	Healthy	-	-	-	Belgium	doi: 10.1016/j.vetmic.2005.10.023.
CP18	Broiler	Healthy	-	-	-	Belgium	doi: 10.1016/j.vetmic.2005.10.023.
CP20	Broiler	Healthy	-	-	-	Belgium	doi: 10.1016/j.vetmic.2005.10.023.
CP21	Broiler	Healthy	-	-	-	Belgium	doi: 10.1016/j.vetmic.2005.10.023.
CP22	Broiler	Healthy	-	-	-	Belgium	doi: 10.1016/j.vetmic.2005.10.023.
CP23	Broiler	Healthy	+	+	+	Belgium	doi: 10.1016/j.vetmic.2005.10.023.
CP24	Broiler	Healthy	-	-	-	Belgium	doi: 10.1016/j.vetmic.2005.10.023.
CP25	Broiler	Healthy	-	-	-	Belgium	doi: 10.1016/j.vetmic.2005.10.023.
CP27	Broiler	Healthy	-	-	-	Belgium	doi: 10.1016/j.vetmic.2005.10.023.
CP28	Broiler	NE	+	+	+	Belgium	doi: 10.1016/j.vetmic.2005.10.023.
CP37	Broiler	NE	+	+	+	Belgium	doi: 10.1016/j.vetmic.2005.10.023.
CP38	Broiler	NE	+	+	+	Belgium	doi: 10.1016/j.vetmic.2005.10.023.
CP43	Broiler	NE	-	-	-	Belgium	doi: 10.1016/j.vetmic.2005.10.023.
CP56 (JIR12037)	Broiler	NE	+	+	+	Belgium	doi: 10.1016/j.vetmic.2005.10.023.
CP58	Broiler	NE	+	+	+	Belgium	doi: 10.1016/j.vetmic.2005.10.023.
CP60	Broiler	NE	+	+	+	Belgium	doi: 10.1016/j.vetmic.2005.10.023.
CP61	Broiler	NE	+	+	+	Belgium	doi: 10.1016/j.vetmic.2005.10.023.
97.78718-2 (D2)	Broiler	NE	+	+	+	Denmark	Pedersen K., pers. Com.; doi.org/10.3389/fcimb.2021.645248
99.63206-34 (JIR12033) (D3)	Broiler	NE	+	+	+	Denmark	doi: 10.1051/vetres/2009069
00.82196-2 (D4)	Broiler	NE	-	-	-	Denmark	Pedersen K., pers. Com.; doi.org/10.3389/fcimb.2021.645248
30100-1-B1 (D5)	Broiler	NE	+	+	+	Denmark	Pedersen K., pers. com.
200302-1-1-Ba (D6)	Broiler	NE	+	+	+	Denmark	doi: 10.1051/vetres/2009069
75-659481-1(D7)	Broiler	NE	+	+	+	Denmark	Pedersen K., pers. com.
70292-4 (D8)	Broiler	NE	+	+	+	Denmark	Pedersen K., pers. Com.; doi.org/10.3389/fcimb.2021.645248
75.65603-1 (D9)	Broiler	NE	-	-	-	Denmark	Pedersen K., pers. com.
75.65603-2 (JIR 12036) (D10)	Broiler	NE	-	-	-	Denmark	Pedersen K., pers. com.
75.65948-1 (D11)	Broiler	NE	+	+	+	Denmark	doi: 10.1051/vetres/2009069
75.65948-6 (D12)	Broiler	NE	+	+	+	Denmark	Pedersen K., pers. com.
97.71994-2 (D13)	Broiler	NE	+	+	+	Denmark	Pedersen K., pers. Com.; doi.org/10.3389/fcimb.2021.645248
98.73920-13 (D14)	Broiler	NE	-	-	-	Denmark	Pedersen K., pers. com.
00.71842-1 (D15)	Broiler	NE	+	+	+	Denmark	Pedersen K., pers. Com.; doi.org/10.3389/fcimb.2021.645248
EHE-NE1 (JIR4857)	Broiler	NE	-	-	-	Australia	doi: 10.1186/s13567-020-00825-6
EHE-NE5 (JIR4860)	Broiler	NE	+	+	+	Australia	doi: 10.1128/JCM.42.3.1345-1347.2003
EHE-NE15 (JIR4866)	Broiler	NE	+	+	+	Australia	doi: 10.1128/JCM.42.3.1345-1347.2003

EHE-NE18 (JIR4869)	Broiler	NE	+	+	+	Australia	doi: 10.1128/JCM.42.3.1345-1347.2003
EHE-NE20 (JIR4870)	Broiler	NE	+	+	+	Australia	doi: 10.1128/IAI.00806-06
NAG-NE23 (JIR4873)	Broiler	NE	-	-	-	Australia	doi: 10.1128/JCM.42.3.1345-1347.2003
NAG-NE24 (JIR4874)	Broiler	NE	-	-	-	Australia	doi: 10.1128/JCM.42.3.1345-1347.2003
UNK-NE30 (JIR12058)	Broiler	NE	+	+	+	Australia	doi: 10.1051/vetres/2009069
EHE-NE32 (JIR12071)	Broiler	NE	-	-	-	Australia	doi: 10.1051/vetres/2009069
JGS4096	Broiler	NE	-	-	-	USA	G. Songer, pers. com.
JGS4100	Broiler	NE	+	+	+	USA	G. Songer, pers. com.; doi.org/10.3389/fcimb.2021.645248
JGS4102	Broiler	NE	+	+	+	USA	G. Songer, pers. com.
JGS4104	Broiler	NE	-	-	-	USA	G. Songer, pers. com.
JGS4120	Broiler	NE	-	-	-	USA	G. Songer, pers. com.
JGS4121	Broiler	NE	-	-	-	USA	G. Songer, pers. com.; doi.org/10.3389/fcimb.2021.645248
JGS4122	Broiler	NE	-	-	-	USA	G. Songer, pers. com.
JGS4123	Broiler	NE	+	+	+	USA	G. Songer, pers. com.
JGS4125	Broiler	NE	-	-	-	USA	G. Songer, pers. com.
JGS4126	Broiler	NE	-	-	-	USA	G. Songer, pers. com.
PK805	Broiler	NE	-	-	-	Belgium	this study
PL687	Broiler	NE	-	-	-	Belgium	this study
PK22	Broiler	NE	-	-	-	Belgium	this study
PM69	Broiler	NE	+	+	+	Belgium	this study
PM100	Broiler	NE	-	-	-	Belgium	this study
PK403	Broiler	NE	-	-	-	Belgium	this study
PK406	Broiler	NE	-	-	-	Belgium	this study
PK407	Broiler	NE	-	-	-	Belgium	this study
PJ660	Broiler	NE	-	-	-	Belgium	this study
S2	Broiler	NE	-	-	-	Denmark	L. Abildgaard, pers.com.
S13	Broiler	NE	+	+	+	Denmark	L. Abildgaard, pers.com.
S27	Broiler	NE	-	-	-	Denmark	L. Abildgaard, pers.com.
S36	Broiler	NE	+	+	+	Denmark	L. Abildgaard, pers.com.
T2	Broiler	NE	-	-	-	Denmark	L. Abildgaard, pers.com.
T19	Broiler	NE	+	+	+	Denmark	L. Abildgaard, pers.com.
CP72	Layer pullet	NE	-	-	-	Belgium	doi.org/10.1080/03079457.2020.1772460
CP97	Layer pullet	NE	-	-	-	Belgium	this study
CP117	Layer pullet	NE	-	-	-	Belgium	this study
CP148	Layer pullet	NE	-	-	-	Belgium	this study
CP199	Layer pullet	Healthy	-	-	-	Belgium	this study
CP200	Layer pullet	Healthy	-	-	-	Belgium	this study
CP203	Layer pullet	Healthy	-	-	-	Belgium	this study
BCP62	Bovine	Bovine enterotoxaemia	-	-	-	Belgium	doi: 10.1016/j.jcpa.2012.11.237
BCP20	Bovine	Healthy calf (abomasal ulcer)	-	-	-	Belgium	doi: 10.1016/j.jcpa.2010.02.004
MLP8	Sheep	/	-	-	-	Switzerland	H. Posthaus, pers. com.
MLP144	Deer	/	-	-	-	Switzerland	H. Posthaus, pers. com.
JF3721	Pig	Piglet with necrotizing enteritis	-	-	-	Switzerland	doi: 10.1128/IAI.01284-09
JIR325	Human	Gas gangrene	-	-	-	Australia	doi: 10.1086/514022
7442	Bovine	Bovine enterotoxaemia	-	-	-	Belgium	this study
BCP513	Bovine	Healthy calf	-	-	-	Belgium	doi: 10.1186/1746-6148-10-32
BCP447	Bovine	Healthy calf	-	-	-	Belgium	doi: 10.1016/j.jcpa.2012.11.237
BCP730	Bovine	Bovine enterotoxaemia	-	-	-	Belgium	doi: 10.1186/1746-6148-10-32
BCP740	Bovine	Healthy calf	-	-	-	Belgium	doi: 10.1186/1746-6148-10-32
BCP747	Bovine	Healthy calf	-	-	-	Belgium	doi: 10.1186/1746-6148-10-32
BCP783	Bovine	Ruminating cattle	-	-	-	Belgium	doi: 10.1186/1746-6148-10-32
BCP815	Bovine	Ruminating cattle	-	-	-	Belgium	doi: 10.1186/1746-6148-10-32
BCP820	Bovine	Ruminating cattle	-	-	-	Belgium	doi: 10.1186/1746-6148-10-32
BCP256	Bovine	Bovine enterotoxaemia	-	-	-	Belgium	doi: 10.1016/j.jcpa.2012.11.237
BCP311	Bovine	Healthy calf	-	-	-	Belgium	doi: 10.1186/1746-6148-10-32
BCP588	Bovine	Bovine enterotoxaemia	-	-	-	Belgium	doi: 10.1186/1746-6148-10-32
BCP334	Bovine	Healthy calf	-	-	-	Belgium	doi: 10.1016/j.jcpa.2012.11.237
BCP506	Bovine	Healthy calf	-	-	-	Belgium	doi: 10.1016/j.jcpa.2012.11.237
BCP510	Bovine	Bovine enterotoxaemia	-	-	-	Belgium	doi: 10.1016/j.jcpa.2012.11.237
MLP98	Sheep	Healthy	-	-	-	Switzerland	H. Posthaus, pers. com.
DCP1.1	Dog	Healthy	-	-	-	Belgium	This study
DCP1.2	Dog	Healthy	-	-	-	Belgium	This study
ECP1	Horse	Healthy	-	-	-	Belgium	This study
ECP2	Horse	Healthy	-	-	-	Belgium	This study
ECP3	Horse	Healthy	-	-	-	Belgium	This study
ECP4	Horse	Healthy	-	-	-	Belgium	This study
ECP5	Horse	Healthy	-	-	-	Belgium	This study
SCP1	Sheep	Healthy	-	-	-	Belgium	doi: 10.1186/1746-6148-10-32
SCP2	Sheep	Healthy	-	-	-	Belgium	doi: 10.1186/1746-6148-10-32
SCP3	Sheep	Healthy	-	-	-	Belgium	doi: 10.1186/1746-6148-10-32
SCP4	Sheep	Healthy	-	-	-	Belgium	doi: 10.1186/1746-6148-10-32
SCP5	Sheep	Healthy	-	-	-	Belgium	doi: 10.1186/1746-6148-10-32
GCP1	Goat	Healthy	-	-	-	Belgium	This study
GCP2	Goat	Healthy	-	-	-	Belgium	This study
GCP3	Goat	Healthy	-	-	-	Belgium	This study
