# Supplementary material for: Clostridium perfringens chitinases, key enzymes during early stages of necrotic enteritis in broiler chickens
Source: PLoS Pathog. 2024 Sep 16;20(9):e1012560. doi: 10.1371/journal.ppat.1012560 (PMC11426533; doi:10.1371/journal.ppat.1012560)
Supplement: S6 Table — (PDF) [file ppat.1012560.s006.pdf]

S6 Table: Variants detected in CP56 chitinase mutants.

Strain(s)	Variant Type	Variant (DNA)	Variant (Protein)	OrfID	Gene	Gene product	Comments
CP56Δ*chiA*1	missense_variant	c.380G>T	p.Arg127Ile	FHMOEFBK_00914	adhE_3	Aldehyde-alcohol dehydrogenase	
	missense_variant	c.1546G>T	p.Ala516Ser	FHMOEFBK_01293	ykoD	Putative HMP/thiamine import ATP-binding protein YkoD	6% of reads correct variant detected
	missense_variant	c.226G>T	p.Ala76Ser	FHMOEFBK_01743	grpE_1	Protein GrpE	
	missense_variant	c.451G>T	p.Ala151Ser	FHMOEFBK_02364	mccF	Microcin C7 self-immunity protein MccF	
	missense_variant	c.99G>T	p.Lys33Asn	FHMOEFBK_03168		Hypothetical protein	
CP56Δ*chiA2*	missense_variant	c.836C>T	p.Thr279Ile	FHMOEFBK_01691	topB_2	DNA topoisomerase 3	
	missense_variant	c.458C>A	p.Ser153Tyr	FHMOEFBK_01878		hypothetical protein	
	missense_variant	c.179G>T	p.Gly60Val	FHMOEFBK_02207	csh	Carbon disulfide hydrolase	
	missense_variant	c.355C>A	p.Gln119Lys	FHMOEFBK_02477		hypothetical protein	
	stop_gained	c.1090G>T	p.Glu364*	FHMOEFBK_02990		hypothetical protein	total length: 1137,but shorter
	missense_variant	c.703G>T	p.Asp235Tyr	FHMOEFBK_03023		Arylsulfatase	
	missense_variant	c.998C>A	p.Ala333Glu	FHMOEFBK_03131	brnQ_3	Branched-chain amino acid transport system 2 carrier protein	
CP56Δ*chiB1*	missense_variant	c.525C>A	p.Ser175Arg	FHMOEFBK_00530	glpF_1	Glycerol uptake facilitator protein	
	missense_variant	c.830G>T	p.Arg277Ile	FHMOEFBK_01764		Cobalt-dependent inorganic pyrophosphatase	
CP56Δ*chiB2*	missense_variant	c.1852G>T	p.Asp618Tyr	FHMOEFBK_00810		hypothetical protein	
	missense_variant	c.1616G>T	p.Arg539Ile	FHMOEFBK_00922		hypothetical protein	
	missense_variant	c.599C>A	p.Ala200Glu	FHMOEFBK_01347	ntpA	V-type sodium ATPase catalytic subunit A	31% of reads correct variant detected
	missense_variant	c.117G>T	p.Met39Ile	FHMOEFBK_01431	rpmF	50S ribosomal protein L32	
	missense_variant	c.706G>A	p.Asp236Asn	FHMOEFBK_01947		hypothetical protein	
	missense_variant	c.457G>T	p.Ala153Ser	FHMOEFBK_01949		hypothetical protein	39% of reads correct variant detected
	missense_variant	c.895G>T	p.Val299Leu	FHMOEFBK_02166		hypothetical protein	
	missense_variant	c.3159G>T	p.Lys1053Asn	FHMOEFBK_02803	rpoC	DNA-directed RNA polymerase subunit beta	
