# Supplementary material for: Clostridium perfringens chitinases, key enzymes during early stages of necrotic enteritis in broiler chickens
Source: PLoS Pathog. 2024 Sep 16;20(9):e1012560. doi: 10.1371/journal.ppat.1012560 (PMC11426533; doi:10.1371/journal.ppat.1012560)
Supplement: S7 Table — (PDF) [file ppat.1012560.s007.pdf]

S7 Table: Variants detected in CP56 chitinase mutants and BLASTp results with predicted impacts.

Strain(s)	OrfID	Genbank accession	% identity	Description	Extra info
CP56Δ*chiA1*	FHMOEFBK_00914	WP_110083220.1	100	Iron-containing alcohol dehydrogenase	Catalyses oxidation of ethanol to acetaldehyde
	FHMOEFBK_01293	WP_003456102.1	100	ABC transporter ATP-binding protein	Regulating the import and export of substances across plasma membranes
	FHMOEFBK_01743	WP_003455172.1	100	Nucleotide exchange factor GrpE	Regulation of protein folding, heat shock
	FHMOEFBK_02364	WP_078209983.1	100	S66 peptidase family protein	LD-carboxypeptidases, bacterial antibiotic resistance
	FHMOEFBK_03168	WP_003450903.1	100	DUF1667 domain-containing protein	Potential metal-binding proteins
CP56Δ*chiA2*	FHMOEFBK_01691	EGT5618054.1	99,86	Type IA DNA topoisomerase	Function in transcription and DNA replication
	FHMOEFBK_01878	ELC8349289.1	97,78	Zn-dependent exopeptidase M28	Bacterial leucyl aminopeptidases, release N-terminal AA residues from polypeptides
	FHMOEFBK_02207	WP_011009793.1	100	Carbonic anhydrase	pH regulation
	FHMOEFBK_02477	WP_003456782.1	100	Peptidylprolyl isomerase	Interconverts the cis and trans isomers of peptide bonds with the amino acid proline
	FHMOEFBK_02990	WP_004458069.1	100	Mechanosensitive ion channel family protein	Osmotic balance
	FHMOEFBK_03023	WP_110083405.1	100	Arylsulfatase	Cleavage sulphate-containing fats
	FHMOEFBK_03131	WP_003458178.1	100	Branched-chain amino acid transport system 2 carrier	Diffusion of sugars, amino acids, and nucleosides across membrane
CP56Δ*chiB1*	FHMOEFBK_00530	WP_004457307.1	100	MIP/aquaporin family protein	Aqueous pores, passive transport of their solute(s) across the membrane
	FHMOEFBK_01764	WP_004457849.1	100	Putative manganese-dependent inorganic diphosphatase	Controls biosynthetic reactions that produce Ppi, RNA synthesis
CP56Δ*chiB2*	FHMOEFBK_00810	VTQ57712.1	99,8	CoA-substrate-specific enzyme activase	Provide a reactive electron that their cognate enzyme requires for activity
	FHMOEFBK_00922	WP_110083215.1	100	DUF4091 domain-containing protein	Domain with uncharacterised function, often conserved in N-acetylgalactosaminidases
	FHMOEFBK_01347	WP_003449648.1	100	V-type ATP synthase subunit A	Produces ATP from ADP in the presence of a proton gradient across the membrane
	FHMOEFBK_01431	WP_003449428.1	100	50S ribosomal protein L32	Binds/regulates splicing and translation of transcript
	FHMOEFBK_01947	WP_224391477.1	100	Alpha/beta fold hydrolase	Catalyze bond cleavages by reaction with water, break down nutrients for digestion
	FHMOEFBK_01949	WP_078209964.1	100	IS110 family transposase	Binding/catalysation transposon to DNA
	FHMOEFBK_02166	WP_057230598.1	100	GH25 family lysozyme	Hydrolyzing glycosidic bonds in peptidoglycans, function debatable
	FHMOEFBK_02803	WP_224361666.1	99,92	DNA-directed RNA polymerase subunit beta'	RNA synthesis
