# Supplementary figures and images for: Clostridium perfringens chitinases, key enzymes during early stages of necrotic enteritis in broiler chickens
Source: PLoS Pathog. 2024 Sep 16;20(9):e1012560. doi: 10.1371/journal.ppat.1012560 (PMC11426533; doi:10.1371/journal.ppat.1012560)

S1 Fig: Overview of DNA sequence of chitinases ChiA and ChiB with annotated features and primers

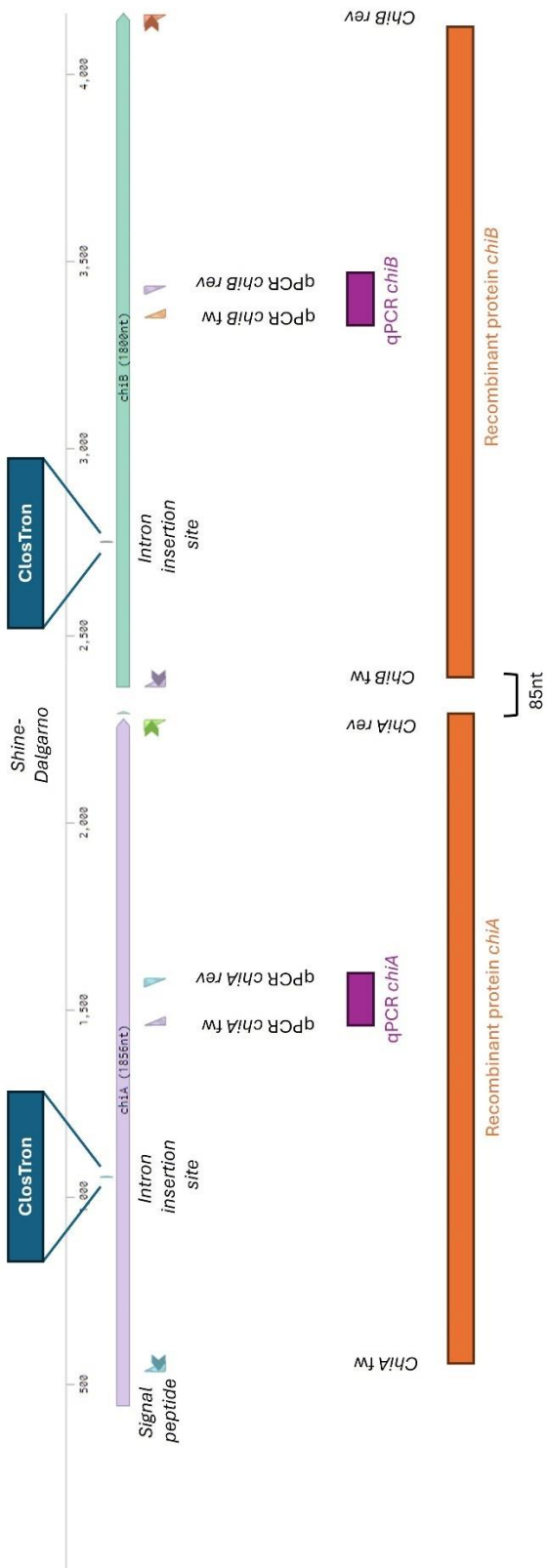

Supplement: S1 Fig — (PDF) [file ppat.1012560.s008.pdf]
