# Supplementary material for: Clostridium perfringens chitinases, key enzymes during early stages of necrotic enteritis in broiler chickens
Source: PLoS Pathog. 2024 Sep 16;20(9):e1012560. doi: 10.1371/journal.ppat.1012560 (PMC11426533; doi:10.1371/journal.ppat.1012560)

S2 Fig: Expression of chitinase genes using qPCR

The expression of *chiA* and *chiB* on RNA level was assessed using qPCR. *C. perfringens* strain CP56 (Belgian Coordinated Collections of Microorganisms LMG 33101) was grown in either nutrient rich medium (Brain heart infusion broth, BHI; Thermo Fisher) or nutrient poor medium ((50% tryptic soy broth, 25% nutrient broth and 25% peptone water) supplemented with 5% chicken intestinal mucus. RNA was extracted during the exponential growth of the bacterial culture, using the Aurum Total RNA mini kit (Bio-Rad). DNA was removed using the Turbo DNA-free kit (Invitrogen). cDNa was synthesized using the iScript cDNA synthesis kit (Bio-Rad). The expression of the chitinase genes was assessed using a SYBRgreen qPCR assay. Each 12 µl qPCR reaction consisted of 2µl template cDNA (30ng), 6 µl SensiMix™ SYBR1 & Fluorescein Kit (Bioline), 0.5 µM forward primer and 0.5 µM reverse primer. Cycling was performed on a real-time PCR thermal cycler (Biorad) and conditions were as follows: 95˚C for 10 min, followed by 40 cycles of 95˚C for 45 s and 60˚C for 1 min. The fluorescent products were detected at the last step of each cycle. Data was analysed using the qBase+ software. The qPCR assay was performed on three biological replicates. Normality was checked using the Shapiro-Wilk test. Statistical analysis was performed using a Paired one-tailed t-test provided by the GraphPad Prism software. Bars indicate the means with their respective standard deviations.

rpoA: FW 5’-acatcattagcgttgtcagttaaag-3’ , REV 5’-gaggttatggaataactcttggtaatg-3’

chiB: FW 5’-gatgcagacctttctccaacgc-3’, REV 5’-ccatacaccaccagctcttcct-3’

chiA: FW 5’-gggtgggaaaatgttcaaggtgg-3’, REV 5’-gcaccagccggttctaaaactc-3’

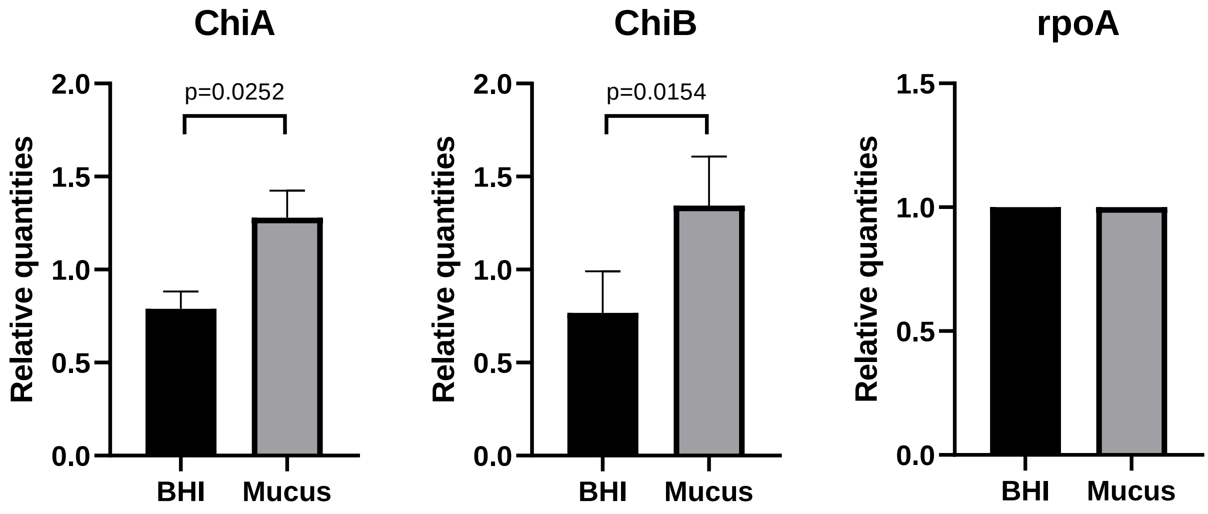

Supplement: S2 Fig — (PDF) [file ppat.1012560.s009.pdf]
