# Supplementary material for: Clostridium perfringens chitinases, key enzymes during early stages of necrotic enteritis in broiler chickens
Source: PLoS Pathog. 2024 Sep 16;20(9):e1012560. doi: 10.1371/journal.ppat.1012560 (PMC11426533; doi:10.1371/journal.ppat.1012560)

**S3 Fig: Structure based alignments of *C. perfringens* ChiA and ChiB with related chitinases.** Top) Structure based sequence alignment of *C. perfringens* ChiA with *C. violaceum* chitinase (pdb 4tgx) and *C. perfringens* ChiB with *S. marcescens* ChiA (pdb 7fd6). Bottom) Cartoon representation of the aligned structures. Alignments were performed with GTalign (Margelevicius, M. GTalign: High-performance protein structure alignment, superposition, and search. bioRxiv 2023.12.18.572167; (2023). doi: https://doi.org/10.1101/2023.12.18.572167) with options -s0 --pre-score=0.1 –depth=0. In grey, parts of the proteins rejected from the alignment.

```
                   GH18-TIM barrel
Query:    34 YKQGGAEQLANGSRRRIIGYFTSWRTGKDGSPAYLASDIPWSKLTHINYAFAHVDG-SNKLSVNETAPGNPATDMSWP-- 110
             +     ++ +      R+++GYF++W+++     ++++++D++W++LTHI+Y+FA+VD+ +NK+++    +  ++A++++++
ChiA :    47 S----EL-N-----RKLVGYFPEWAYSSEAQGYFNVTDLQWDSLTHIQYSFAMVDPSTNKITLSN-K--HAAIEEDFSEF 113
struct                     eeeeeee h h   ht     th     h     ee    eee  h     ee        hhhh      t

Query:   111 GVAG----AEMDASLPYKGHFNLLTQYKRKYPGVKTLISVGGWAETGGYFDANGKRVASGGFYSMTVNADGTVNQAGINA 186
              +++    +E+D+SLPYKGHFN+L+++K++YP+V++LISVGGW++T+                 +FY+M++       ++++IN+
ChiA :   114 DLNYNGKKIELDPSLPYKGHFNVLQTMKKNYPDVSLLISVGGWTGTR-------------CFYTMID------TDNRINT 174
struct       eee ht eee        ethhhhhhhhhhh      eeeee    th                    hhhhhh         thhhhh

                              * * *
Query:   187 FSDSAVAFLRKYGFDGVDIDFEYPTSMNNAGNPLDWTFSNARLGSLNKGYVALLQTLRDRLDRAAAQDGRYYQITAAVPA 266
             F+DS+V+F+RKYGFDGVDIDFEYP+S++++GNP+D+++S++R+++LN++Y++L++TLR+++D+A+++DG++Y++TAAV+A
ChiA :   175 FADSCVDFIRKYGFDGVDIDFEYPSSTSQSGNPDDFDLSEPRRTKLNERYNILIKTLREKIDMASKEDGKEYLLTAAVTA 254
struct       hhhhhhhhhhht     eeee               t th   hhhhh   h   hhhhhhhhhhhhhhhhhhhht   eeeeeeeee

Query:   267 SGYLLRGMETFQGLKYLDFVNVMSYDLHGAWNRFVGPNAALYDDGKDAELAFWNVYSTPQYGNIGYLNTDWAYHYYRGGL 346
             S+++L+G+++++++KYLDF+++MSYD+HG+WN++V+++A++Y++++D+E++   ++        +   +++L++DWAY+YYRG+L
ChiA :   326 SPWVLGGISDNTYAKYLDFLSIMSYDYHGGWNEYVEHLAGIYPNKEDRETV--TQ-----I--MPTLCMDWAYRYYRGVL 371
struct        thhhh      hhh     ee          th     e e   e           h      t                thhhhhhh ht

Query:   347 PASRVNMGVPYYTRGWKNVSGGSNGLWGSSVGSNCPAGLTECGDGAVGIDNIWHDLDDSG-KEIPGGSNPMWHAKNLEKG 425
             PA++++MG+PYYTRGW+NV+GG+NGL+GSS+                ++A+G++NI++D+++++ +++P+G+NP+WH++NL+++
ChiA :   372 PAEKILMGIPYYTRGWENVQGGINGLHGSSK------------TPASGKYNILGDDLNNDGVLEPAGANPLWHVLNLMEQ 393
struct         th  eeeee  eeee       tht   ee                 thh     e  t t  e      e thhhhhhhhh

Query:   426 LAGSYLAAYGIDPTLPINQLTGSYQRNYNGALAAPWLWNAGKKVFLSTEDEQSIAQKAAWIDANNVGGVMFWELAGDYDW 505
                                   +++++++++++++P++W+++KKVF+S+E+E+SI++++++I+++N+GG+++W+++GDY++
ChiA :   394 -------------------D-PNLKVYWDEISKVPYVWQNDKKVFVSFENEKSIDARLEYIQNKNLGGALIWVMNGDYGL 453
struct                                  eeeee ht  eeeee ht eeeeee thhhhhhhhhhht    ee    h  e

                                GH18-TIM barrel          CBD
Query:   506 KAQRNNG-----QGEYFIGTTLTSLLYNTFSQPPKV-S-----A--PTAAIDVGFSLGGFKLGDQNYPINPKLTIVNRSQ 572
             +  ++ g      g+Y++G+TLT++L++++++++            +  ++++I V+++++G+     +++++++++++I+N
ChiA :   454 NPNYVEGSNKINEGKYTFGDTLTKRLSQGLKKMGV-CNKTPDDLNISLEPINVDVKFNGKY---DHPNYTYSIDITNYTD 529
struct           ee      th          hhhhhhhhhhht   e  ee  thh    e   eeeeeeee     tt eeeeeeeee

Query:   573 TTLPGGTEFQFDVPTSAPANIADQSGFGLKVVSAGHSGSNVGGLKGDFNRVSVKLPSWQSLGAGQSVTLDVVYYLPISGP 652
               ++GG++++FD+P+SA+ +++++ G++ ++V+++            GDFN++++++++WQ++    +++T++++++L++SG+
ChiA :   530 KEIKGGWNVSFDLPKSAV-FKSSW-GGT-YSVTDN-----------GDFNTITLTSGAWQNIAPNSTITVQGMIGLCFSGI 596
struct         ee t  eeeeee th e e     t e eeeee              tt eeeeeee th ee tt eeeeeeee  e   e

                                 CBD
Query:   653 SHYTVGLNGKTYAIRDEAPYLPYLR 677
             +++T++    ++++
ChiA :   597 RNVTFN----GMNPIG--------N 609
struct         ee h    t e e

TM-score (Refn./Query) = 0.89706 / 0.74990, d0 (Refn./Query) = 8.35 / 9.02,  RMSD = 2.60 A
Identities = 209/665 (31%), Matched = 511/665 (76%), Gaps = 123/665 (18%)
```

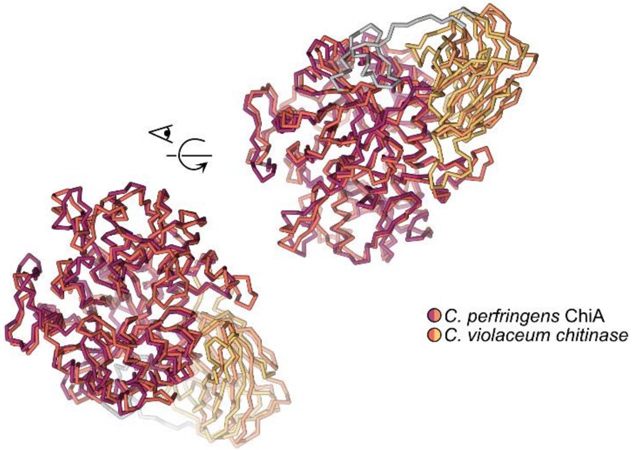


```
         CBD...   GH18-TIM barrel
Query: 134 GKVVGSYFVEWGVYG-R-NFTVDKIPAQNLTHLLYGFIPICGGNGINDSLKEIEGSFQALQRSCQGREDFKVSIHDPFAA 211
           ++++++Y+++W+++G + NF++++IPA++LTHL+++F+++++                          ++++++D++AA
ChiB :  25 KLRNVMYYGDWSIWGGQGNFYPKDIPADKLTHLNFAFMDFNS--------------------------SGELIYCDKDAA 78
struct       eeeeee th  ttht  e th    h    ee    eee t                          t  eee    hhh

                                                                                      *
Query:   212 LQKAQKGVTAW-DDPYKGNFGQLMALKQAHPDLKILPSIGGWTLSDPFFF-MGDKVKRDRFVGSVKEFLQTWKFFDGVDI 289
             +++++++ +++  D+++G+++++++LK+++P+LKI++S+GGW++S++F++ ++++++R++FV++V++F+++++ +D+VDI
ChiB :    79 IGHPLGNLGVTYGDVNGGILNAFQVLKSENPNLKIGVSLGGWSKSGDFSTIAATPSIRAKFVENVMKFIKYTN-MDFVDI 237
struct       t    t     t  t   thhhhhhhhhh    eeeee    hh    hhhhhhh   hhhhhhhhhhhhhhhhht      eee

             * *
Query:   290 DWEFPGGKG-A---------NPNLGSPQDGETYVLLMKELRAMLDQLSAETGRKYELTSAISAGKDKID-KVAYNVAQNS 358
             DWE+PG+++ +         + +++S++D+E+Y+LL+++L+++L++++++E+G++YEL++A++AG++KI+ ++++++++N+
ChiB :   182 DWEYPGDYREPDKTDNINDEGTPNASAGDKENYILLLQDLKEALNKQGKELGKVYELSVALPAGVSKIEKGIDVDKLFNI 237
struct       e       ee     hh t  e          thhhhhhhhhhhhhhhhhhhhhhht   eeeeeeeee   thhhhht    hhhhht

Query:   359 MDHIFLMSYDFYGAFDLKNLGHQTALNAPAWKPD--TAYTTVNGVNALLTQGVKPGKIVVGTAMYGRGWTGVNGYQ--N- 433
             +D++++M+YD++GA++ +++GHQTAL+++++p   ++++++++V+++++QG++++KIVVG+A+Y+RGW++V+ ++  +
ChiB :   238 VDFANIMTYDMAGAWS-TTSGHQTALYTNPNAPEEYKGLSVDESVKYYISQGAEREKIVVGAAYYTRGWEQVSDKGTDPN 316
struct         ee         th    e e        t    th        thhhhhhhhhh    th   eeeee   eeee   e         h

Query:   434 -NIPFTGTAT----------GPVKGTW------E--NG-IVDYRQIASQFM-SGEWQYTYDATAEAPYVFKPSTGDLITF 492
              +++F+++A+           +++++++      +  +G +++Y+++  ++    +++++D++A+APY+++++TG+++T+
ChiB :   317 NPGLFGEAAVVNKDADLSPTPGALNEAPMKNGEGGRAGGVWGYNALDKLKSKYTGLKEYWDDSAKAPYLYNSETGAFFTY 396
struct                e     e tt          t    thh     e    e th    hhhhhh    eeeee   ht   eeee   ht eeeee

                                                       GH18-TIM barrel
Query:   493 DDARSVQAKGKYVLDKQLGGLFSWEIDADN-------GDILNSMNASLGNSAG 538
             D++RS+Q+K+KYV++++LGG+++W++++D+        ++++++++++SL+++
ChiB :   397 DNIRSIQEKAKYVKENNLGGIIGWMASQDATTNSTKRDELTTATKESLFGKED 449 ...CBD
struct       e thhhhhhhhhhhht    eee     th   ee tt     thhhhhhhht

TM-score (Refn./Query) = 0.62946 / 0.66790, d0 (Refn./Query) = 8.40 / 8.19,  RMSD = 2.02 A
Identities = 117/453 (25%), Matched = 364/453 (80%), Gaps = 76/453 (16%)
```

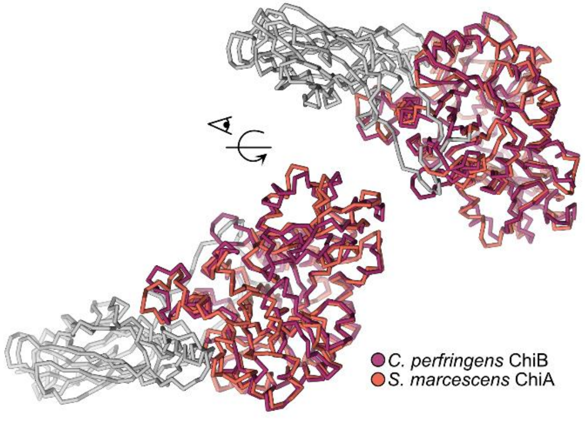

Supplement: S3 Fig — (PDF) [file ppat.1012560.s010.pdf]
