# Supplementary material for: Clostridium perfringens chitinases, key enzymes during early stages of necrotic enteritis in broiler chickens
Source: PLoS Pathog. 2024 Sep 16;20(9):e1012560. doi: 10.1371/journal.ppat.1012560 (PMC11426533; doi:10.1371/journal.ppat.1012560)
Supplement: S4 Fig — (PDF) [file ppat.1012560.s011.pdf]

S4 Fig: NetB activity in culture supernatant of *C. perfringens* strains (wild-type and mutant strain) towards chickens erythrocytes.

The NetB production of the *C. perfringens* strains was evaluated through an haemolytic assay, previously described by Hustá *et al*. In short, 20% culture supernatant was incubated with 2% (v/v) chicken erythrocytes (diluted in HBSS-) in a 96-well plate. After an incubation period of 30 minutes at 37°C, intact red blood cells were pelleted and the released hemoglobin in the supernatant was quantified at OD_{550nm}. A matched non-parametric Friedman test with Dunn' multiple comparison (confidence interval 95%) was performed using GraphPad Prism 8 software. Bars indicate the means with their respective standard deviations. No significant difference in NetB activity between the strains could be detected.

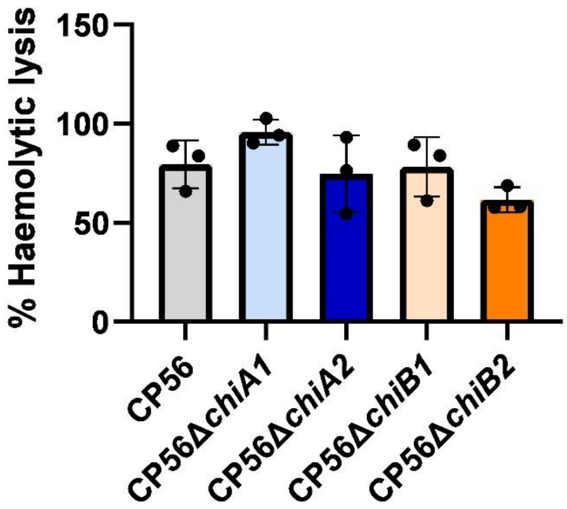

[1] Hustá M, Ducatelle R, Van Immerseel F, Goossens E. A Rapid and Simple Assay Correlates In Vitro NetB Activity with Clostridium perfringens Pathogenicity in Chickens. Microorganisms. 2021 Aug 11;9(8):1708. doi: 10.3390/microorganisms9081708. PMID: 34442787; PMCID: PMC8400579.
