# Supplementary material for: Clostridium perfringens chitinases, key enzymes during early stages of necrotic enteritis in broiler chickens
Source: PLoS Pathog. 2024 Sep 16;20(9):e1012560. doi: 10.1371/journal.ppat.1012560 (PMC11426533; doi:10.1371/journal.ppat.1012560)

S5 Fig: Growth analysis mutant strains in different culture media

Nutrient rich medium: Brain heart infusion broth

Nutrient poor medium: 50% tryptic soy broth, 25% nutrient broth and 25% peptone water

Overnight cultures were 1/1000 diluted in different media. The optical density at 600nm was measured semi-continuously.

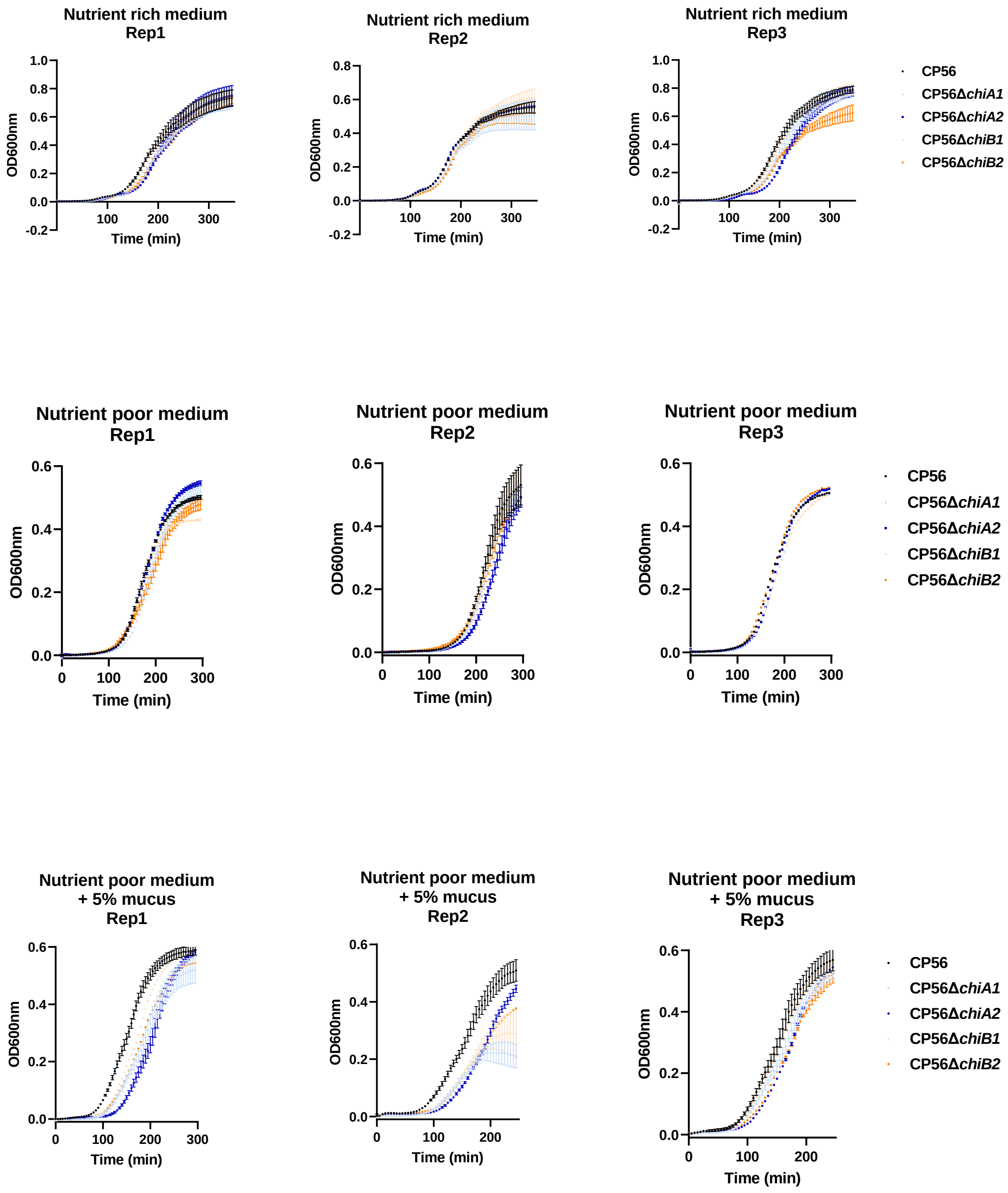

Supplement: S5 Fig — (PDF) [file ppat.1012560.s012.pdf]
