# Supplementary material for: Clostridium perfringens chitinases, key enzymes during early stages of necrotic enteritis in broiler chickens
Source: PLoS Pathog. 2024 Sep 16;20(9):e1012560. doi: 10.1371/journal.ppat.1012560 (PMC11426533; doi:10.1371/journal.ppat.1012560)

S6 Fig: Purity of recombinant ChiA and ChiB using Coomassie staining of SDS-page gel.

The recombinant proteins were extracted from *E.coli* (harbouring the pBAD vector with chitinase insert) using the BugBuster® protein extraction reagent (Merck, Overijse, Belgium) and purified on a Ni-Sepharose column (His GraviTrap, Cytiva, Hoegaarden, Belgium) both according to manufacturer's instructions. Proteins were eluted using PBS containing 500 mM imidazole and 10% glycerol. The eluted solution was dialyzed against PBS containing 10% glycerol to get rid of the residual imidazole fraction. The purity of the proteins was evaluated using SDS-page and Coomassie Brilliant Blue R-250 staining of the gel. Protein concentration was quantified using the Pierce™ BCA Protein Assay Kit (Fisher Scientific, Brussel, Belgium).

From left to right: molecular weight markers, ChiB (2.5 µg/µl), ChiA (5 µg/µl)

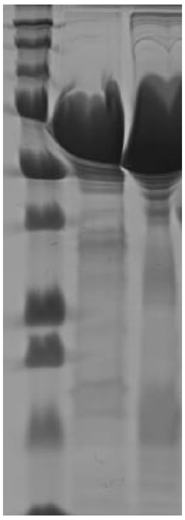

Supplement: S6 Fig — (PDF) [file ppat.1012560.s013.pdf]
