# Supplementary material for: Clostridium perfringens chitinases, key enzymes during early stages of necrotic enteritis in broiler chickens
Source: PLoS Pathog. 2024 Sep 16;20(9):e1012560. doi: 10.1371/journal.ppat.1012560 (PMC11426533; doi:10.1371/journal.ppat.1012560)
Supplement: S1 Data — (PDF) [file ppat.1012560.s014.pdf]

S1 Data: Nucleotide and protein sequence of chitinases ChiA and ChiB

DNA sequence *chiA* (GenBank accession number F8UNI5)

atgaaaaattcaaaattaaaacaaatcttaataacatttcttacaattacattaacttcaagctatcttgtaacatcatcaaatcctatagaaaca
aaagctaaagaaaaatttaaaacaacaaaaattaaaaattcatctgaacttaatagaaagctagtcggatattttccagaatgggcatattcta
gtgaagcacaaggatattttaatgtaacagatttacaatgggattcattaacacatatacaatattcctttgctatggtagatccatctacaaata
aaattactttaagtaataaacatgctgcaattgaagaagatttttcggaatttgatttaaactacaacgggaagaaaatagaattagatccttctt
taccttataaaggacacttcaacgttttacaaactatgaaaaaaaattatccagatgtatcattattaatttctgttggaggttggacaggaacaa
gatgtttctacactatgattgacactgataatagaataaatacatttgcagattcatgtgttgattttatcagaaaatatggatttgat**ggagtaga**
cattgatttt<u>gaa</u>tatccttcatccacaagccaatcagggaatccagacgattttgatctttcagaaccaagaaggacaaaacttaacgaaagat
ataatattttaataaaaactttaagagaaaaaatagatatggcatctaaagaagatggaaaagaatatttattaacagctgctgttactgcttctc
cttgggttttaggcggaatatcggataatacttatgcaaagtacttagatttcttaagcataatgtcatatgactaccacggtggttggaatgaata
tgtagaacacttagctggtatttatcctaataaggaagatagagaaacagtaactcaaataatgccaactttatgtatggattgggcctacagat
attacagaggtgttttgcctgctgaaaaaatattaatgggtatcccttattacacaagagggtgggaaaatgttcaaggtggaattaatggacttc
atggatcaagtaaaactccagcatcaggcaaatataatattttgggagacgatttaaataatgatggagttttagaaccggctggtgcaaatcct
ttatggcatgtattaaatcttatggaacaagatccaaatttaaaagtatattgggatgaaatttctaaagtaccatatgtttggcaaaacgataaa
aaagtattcgtatcatttgaaaatgaaaaatctatagatgcaagattagaatatatacaaaataaaaatcttggtggcgctttaatttgggtaatg
aacggagattacggattaaatccgaattatgtagaaggttcaaataagataaatgaaggtaaatatacttttggagatacattaacgaaaagat
taagccaaggtttaaaaaagatgggagtttgcaataaaacaccagatgacttaaatatatctttagagcctattaatgtagatgttaaatttaatg
gaaagtatgatcatcccaattacacatattcaattgatataacaaattatacagacaaggaaattaaaggtggatggaatgtttcatttgatttac
caaagtctgctgtatttaaatcatcatggggaggcacatatagtgttacagacaatggtgactttaatacaataacattaacttcaggagcttggc
aaaatatagcgcctaactcaacaataacagttcaaggtatgattggattatgcttctctggaataagaaatgtcactttcaatggaatgaatcca
attggtaatgataaataa

- ⇨ Target region ClosTron mutagenesis in bold
- ⇨ Proton donor region underlined

Protein sequence ChiA

MKNSKLKQILITFLTITLTSSYLVTSSNPIETKAKEKFKTTKIKNSSELNRKLVGYFPEWAYSSEAQGYFNVTDLQWDSL
THIQYSFAMVDPSTNKITLSNKHAAIEEDFSEFDLNYNGKKIELDPSLPYKGHFNVLQTMKKNYPDVSLLISVGGWT
GTRCFYTMIDTDNRINTFADSCVDFIRKYGFDGV<u>DIDFE</u>YPSSTSQSGNPDDFDLSEPRRTKLNERYNILIKTLREKID
MASKEDGKEYLLTAAVTASPWVLGGISDNTYAKYLDFLSIMSYDYHGGWNEYVEHLAGIYPNKEDIETVTQIMPTL
CMDWAYRYYRGVLPAEKILMGIPYYTRGWENVQGGINGLHGSSKTPASGKYNILGDDLNNDGVLEPAGANPLW
HVLNLMEQDPNLKVYWDEISKVPYVWQNDKKVFVSFENEKSIDARLEYIQNKNLGGALIWVMNGDYGLNPNYVE
GSNKINEGKYTFGDTLTKRLSQGLKKMGVCNKTPDDLNISLEPINVDVKFNGKYDHPNYTYSIDITNYTDKEIKGGW
NVSFDLPKSAVFKSSWGGTYSVTDNGDFNTITLTSGAWQNIAPNSTITVQGMIGLCFSGIRNVTFNGMNPIGNDK

- ⇨ Domains:

Blue	Predicted signal peptide
Orange	Glycosyl hydrolase 18 domain
Green	Carbohydrate binding domain ChiC

- ⇨ Active site DIDFE (underlined)
  = Substitution of E196 to Q reduces the activity of the enzyme.

DNA sequence *chiB* (GenBank accession number F8UNI4)

gtgaatacaatctctgttaaggctatgagtaaatcttctgatacaactgaaattacttctcaatctactactaagttacgaaatgtaatgtattatg
gtgattggtctatttggggtggacaaggtaacttttatcctaaagatattccagcagataaattaacgcacttaaattttgcattcatggatttcaa
ctcatctggtgaattaatttattgcgataaagatgctgcaataggacatccattaggaaatttgggagtaacctatggagatgttaatggtggaat
attaaatgcttttcaagttttaaaatctgaaaatcctaatttaaagataggagtatctttaggtggatggtcaaagtctggcgatttttcaacaata
gccgcaactccttcaataagagctaaatttgtagaaaacgttatgaaattcattaaatataccaatatggactttgtt**gatattgattgggaatat**
cctggagattacagagaacctgataaaactgacaatataaatgatgaaggaacaccaaatgcaagtgcaggagataaagaaaattatatctt
attattacaagatttaaaggaggctttaaataaacaaggaaaagaattaggaaaagtttatgagctatccgtggcattgccagctggcgtatca
aaaatagaaaaaggaatagacgttgacaagttatttaatatagtagattttgctaatataatgacatatgatatggctggagcttggagtacaac
aagtggacatcaaactgccttatacactaaccctaatgctcctgaagaatataagggactttctgtagatgaaagtgttaaatattatatttcaca
aggtgcagaaagagaaaaaatagttgtaggagcagcatattatacccgtggatgggaacaagtttcagataaaggtacagatccaaataatcc
aggattatttggagaagcagctgtagttaataaagatgcagacctttctccaacgccaggagcacttaatgaggctccaatgaaaaatggtgaa
ggaggaagagctggtggtgtatggggatataatgctttagataaattaaaatctaaatatactggtctaaaagagtattgggatgatagtgctaa
agctccatatttatataattctgaaacaggtgcatttttttacttatgataatataagatcaatccaagaaaaagctaaatatgttaaagaaaataa
tcttggtggtataatcggatggatggcttcacaagatgcaactacaaactcaactaaaagagatgaattaacaactgcaactaaggaatctttat
ttggtaaagaagatttacctaaatatgaaataaaatatactgaaaatgatataacttgtacagtaactccagtaaagcaatcatggggaagtgg
aggagtactaaagatgtcaataacaaacaatgagaaattagatgaatctggtgaagtattatcaacagttgaaacatccgcaaaaacagttaa
aaatatgaaagtatatattaagactgatggaatagcaattactggttcacaatatccagcaggacctgttacaaaagaaggagattactatgtta
tagattttggtaagatatcagatggtaagctaatgaaagcaggaataacctttacttttgatttaaatcttgataaagcaattgaagatactaata
atattataagcattgaagtatctcaaagaatgtatcaaacatctcctgaattcaatagacaaacaatttgggaaaatacaaattcatag

- ⇨ Target region ClosTron mutagenesis in bold
- ⇨ Proton donor region underlined

Protein sequence ChiB

MNTISVKAMSKSSDTTEITSQSTTKLRNVMYYGDWSIWGGQGNFYPKDIPADKLTHLNFAFMDFNSSGELIYCDK
DAAIGHPLGNLGVTYGDVNGGILNAFQVLKSENPNLKIGVSLGGWSKSGDFSTIAATPSIRAKFVENVMKFIKYTN
MDFVDIDWEYPGDYREPDKTDNINDEGTPNASAGDKENYILLLQDLKEALNKQGKELGKVYELSVALPAGVSKIEK
GIDVDKLFNIVDFANIMTYDMAGAWSTTSGHQTALYTNPNAPEEYKGLSVDESVKYYISQGAEREKIVVGAAYYTR
GWEQVSDKGTDPNNPGLFGEAAVVNKDADLSPTPGALNEAPMKNGEGGRAGGVWGYNALDKLKSKYTGLKEY
WDDSAKAPYLYNSETGAFFTYDNIRSIQEKAKYVKENNLGGIIGWMASQDATTNSTKRDELTTATKESLFGKEDLPK
YEIKYTENDITCTVTPVKQSWGSGGVLKMSITNNEKLDESGEVLSTVETSAKTVKNMKVYIKTDGIAITGSQYPAGP
VTKEGDYYVIDFGKISDGKLMKAGITFTFDLNLDKAIEDTNNIISIEVSQRMYQTSPEFNRQTIWENTNS

- ⇨ Domains:

  Orange Glycosyl hydrolase 18 domain

  Green Carbohydrate binding domain

- ⇨ Active site DIDWE (underlined)

  = Substitution of E160 to D reduces the activity of the enzyme.
